# Unraveling Charge Transport in Heterostructured Nanomotors for Efficient Photocatalytic Motion

**DOI:** 10.1021/acs.nanolett.5c02177

**Published:** 2025-06-16

**Authors:** Yufen Chen, Chunyu Li, Rebeca Ferrer Campos, María José Esplandiu, Jordi Fraxedas, Nicoletta Liguori, Katherine Villa

**Affiliations:** † 202569 Institute of Chemical Research of Catalonia (ICIQ), The Barcelona Institute of Science and Technology (BIST), Tarragona E-43007, Spain; ‡ 172281 Institut de Ciències Fotòniques (ICFO), The Barcelona Institute of Science and Technology, Barcelona 08860, Spain; § Departament de Química Física i Inorgànica, Universitat Rovira i Virgili, Tarragona 43007, Spain; ∥ Catalan Institute of Nanoscience and Nanotechnology (ICN2), CSIC and BIST, Campus UAB, Bellaterra, Barcelona 08193, Spain

**Keywords:** photocatalytic nanomotors, heterojunction structure, electron transfer, near ambient pressure X-ray photoelectron spectroscopy, transient absorption spectroscopy

## Abstract

Photocatalytic micro/nanomotors have emerged as promising tools for environmental remediation, biosensing, and targeted delivery. To enhance their light-driven propulsion, significant efforts have focused on engineering semiconductor heterostructures, which promote charge separation. However, a clear understanding of how these architectures govern photocatalytic mechanisms and influence motion performance remains limited. Here, we design a visible light-responsive nanomotor based on a Fe_2_O_3_-Pt-TiO_2_ trilayered heterostructure, combining narrow-bandgap α-Fe_2_O_3_ and wide-bandgap TiO_2_ with an intermediate Pt layer. Remarkably, Fe_2_O_3_-TiO_2_ nanomotors without the Pt layer exhibit only modest propulsion under visible light, whereas the inclusion of Pt significantly enhances their motility. Through advanced techniques, including *in situ* synchrotron radiation-based near-ambient pressure X-ray photoelectron spectroscopy and transient absorption spectroscopy, we reveal that Pt serves as an efficient electron mediator, enabling directional charge transfer across the heterojunction. This study provides fundamental insights into charge transport in multicomponent nanomotors and introduces a rational strategy for designing efficient photoactive systems.

## Graphical abstract

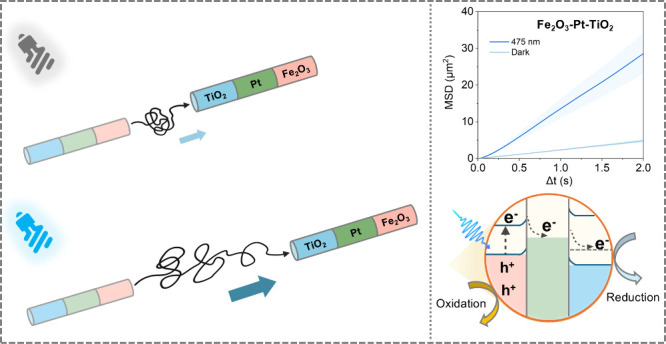

Micro/nanomotors capable of autonomous motion have gained significant attention over the past two decades due to their ability to perform targeted cargo transport, sensing and catalytic reactions in environmental,^1^ analytical and biomedical applications.^2^ Such tiny artificial devices are capable of self-propulsion by converting external energy into mechanical motion.^3–5^ Among the energy resources, light stands out due to its greenness, abundance and remote controllability.^6^ The motion activation of light-driven nanomotors typically relies on photothermal effects, photocatalysis, photon momentum transfer or optical forces.^7^ In particular, photocatalytic nanomotors offer tunable material properties, from inorganic to organic semiconductors,^8^ diverse activation wavelengths, and versatile bandgap engineering strategies for motion modulation.^9,10^

Heterojunction engineering of photocatalytic systems is an attractive approach to enhance charge separation and boost catalytic efficiency.^11–13^ The formation of a built-in electric field at the interface between a semiconductor and a secondary component (metal or another semiconductor) promotes the separation and migration of photogenerated carriers, thereby improving the photocatalytic performance.^14,15^ For instance, semiconductor/metal heterostructured micromotors, such as TiO_2_/Au,^16^ TiO_2_/Au/Pt,^17^ BiOI-Au,^18^ ZnO/Pt,^19^ α-Fe_2_O_3_/Au Janus structures,^20^ have demonstrated efficient motion through self-electrophoresis, where the metal caps serve as the electron acceptors. However, semiconductor/semiconductor heterojunctions remain underexplored in nanomotor design, despite their potential to broaden light absorption, improve catalytic efficiency, and significantly boost propulsion performance. So far, the reported heterojunction-based photocatalytic micro/nanomotors either primarily rely on UV light irradiation,^21–23^ or require complex polarization setups,^24^ limiting their practical implementation. More importantly, a comprehensive understanding of charge transfer mechanisms in heterostructured photocatalytic nanomotors, particularly in relation to their optical properties and motion dynamics, is still lacking.

Herein, we introduce rod-shaped Fe_2_O_3_-Pt-TiO_2_ nanomotors as a visible-light-driven heterojunction system. α-Fe_2_O_3_, with a bandgap of ca. 2.2 eV, is a promising photocatalyst for visible light harvesting, yet its performance is limited by rapid electron–hole recombination. A common strategy to mitigate this drawback involves constructing heterojunctions with complementary semiconductors, which can facilitate charge separation and extend carrier lifetimes.^25^ In our design, α-Fe_2_O_3_ functions as the visible-light absorber due to its narrow bandgap, TiO_2_ serves as the electron transport layer, and an intermediate Pt layer acts as an electron acceptor to facilitate charge separation and enhance motion. Although Fe_2_O_3_/TiO_2_ nanomotors alone showed negligible motion improvement, the incorporation of a Pt interlayer significantly enhanced their propulsion, confirming its role in facilitating electron transfer. To unravel the underlying charge transfer mechanisms in Fe_2_O_3_-Pt-TiO_2_ nanomotors, various cutting-edge techniques have been implemented, including *in situ* near*-*ambient pressure X-ray photoelectron spectroscopy (NAP-XPS) using synchrotron radiation, as well as transient absorption spectroscopy (TAS) measurements. These advanced techniques provide direct insights into electron transfer dynamics and photocatalytic activity, establishing a fundamental framework for the future design of efficient heterojunction-based photocatalytic nanomotors. As proof of concept, the performance of Fe_2_O_3_-Pt-TiO_2_ nanomotors was tested for the photocatalytic degradation of methylene blue (MB) as a model reaction, which is a major contributor to aquatic ecosystem disruption, eutrophication, and aesthetic pollution.^26–29^

The fabrication process of the rod-like α-Fe_2_O_3_-based nanomotors is illustrated in Figure 1a. The nanorod-like α-Fe_2_O_3_ structure was obtained through a facile hydrothermal synthesis, and then the Pt and TiO_2_ layers were further introduced via a sputtering procedure. Field emission scanning electron microscope image (FESEM, Figure 1b) shows that the obtained α-Fe_2_O_3_ nanorods exhibited an average length of ca. 700 ± 20 nm and an average diameter of ca. 116 ± 10 nm. Brunauer-Emmett-Teller (BET) analysis indicates that the specific surface area of as-prepared α-Fe_2_O_3_ nanorods is 17.72 m^2^/g (Table S2). High-resolution transmission electron microscopy (HRTEM) and the corresponding energy-dispersive X-ray (EDX) mapping (Figure 1c) confirm the intended structure of Fe_2_O_3_-Pt-TiO_2_, with Pt forming the middle layer and TiO_2_ in the outer layer, with thicknesses of approximately 10 nm and 10–15 nm, respectively. In addition, we observed that the as-deposited TiO_2_ layer is present in an amorphous phase, as presented in Figures 1d, 1e and S1a. Figure 1f shows that the Fe_2_O_3_-Pt-TiO_2_ nanomotors exhibit a broad absorption peak at around 400–550 nm, due to the optical properties of the α-Fe_2_O_3_ (Figure S1b).^31^ The bandgap of Fe_2_O_3_-Pt-TiO_2_, estimated from its Tauc plot (Figure 1g), indicates that the incorporation of Pt and TiO_2_ does not alter the bandgap energy of the underlying α-Fe_2_O_3_ nanorods (Figure S1c), which remains around 2.1 eV and is favorable for visible light absorption.^32^ For comparison, UV–vis absorption spectra of Fe_2_O_3_-TiO_2_ and Fe_2_O_3_-Pt are also shown in Figures S1d and S1e. The crystalline phases of Fe_2_O_3_-Pt-TiO_2_ nanomotors were analyzed by X-ray diffraction (XRD), and Raman spectroscopy was used to confirm phase composition. XRD patterns (Figures 1h and S1f and S1g) show characteristic reflections of α-Fe_2_O_3_ along with Pt, while no TiO_2_-related peaks were detected due to its low crystallinity. It should be noted that the diffraction patterns from the FTO substrate (SnO_2_) are also observed. Upon calcination, crystallinity improves, revealing the formation of anatase TiO_2_, confirming that TiO_2_ was initially amorphous in the as-prepared Fe_2_O_3_-Pt-TiO_2_ heterostructure (see the Figures S1h and S1i for details). The results also confirm that the iron oxide phase remains unchanged after sputtering. Raman spectrum (Figure 1i) displays peaks at 221 and 496 cm^–1^ (A_1g_ modes), 242, 290, 407, and 608 cm^–1^ (E_g_ modes), and a 661 cm^–1^ peak corresponding to a longitudinal optical phonon mode, all consistent with the hematite structure.^33–35^

**Figure 1:**
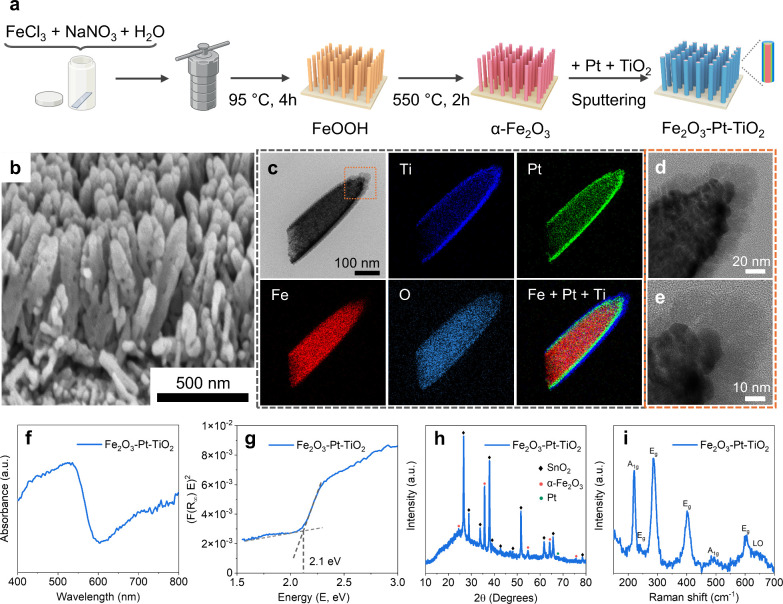
Synthesis and characterization of Fe_2_O_3_-Pt-TiO_2_ nanomotors. (a) Schematic illustration of the synthesis of Fe_2_O_3_-Pt-TiO_2_ nanomotors. (b) FESEM images of as-prepared α-Fe_2_O_3_ nanorods. (c) HRTEM image and the corresponding EDX mapping of Fe_2_O_3_-Pt-TiO_2_ nanomotors. (d) and (e) are the enlarged images of the orange dashed frame in (c). (f) UV–vis absorption spectrum of Fe_2_O_3_-Pt-TiO_2_. (g) Tauc plots of (F­(R_∞_)­E)^2^ versus E­(eV) for Fe_2_O_3_-Pt-TiO_2_. Due to the amorphous nature of TiO_2_, the presence of Pt, and possible interfacial states, the Fe_2_O_3_-Pt-TiO_2_ heterostructure presents additional complexities that complicate the determination of the true absorption onset. To address this, we adopted a more robust approach, which involves fitting two linear regions: one below and one above the absorption edge.^30^ The intersection of these two lines provides an estimate of the optical bandgap. (h) XRD profile of Fe_2_O_3_-Pt-TiO_2_. α-Fe_2_O_3_ (JCPDS 01-087-1165), Pt (JCPDS 00-004-0802). (i) Raman spectrum of Fe_2_O_3_-Pt-TiO_2_.

The motion characterization of bare α-Fe_2_O_3_, Fe_2_O_3_-TiO_2_, Fe_2_O_3_-Pt, and Fe_2_O_3_-Pt-TiO_2_ was evaluated under both dark and blue light (475 nm, 333 mW/cm^2^) conditions. The representative trajectories (Figures 2a and 2b) show that upon light illumination, the motion of bare α-Fe_2_O_3_ and Fe_2_O_3_-TiO_2_ did not significantly differ from their behavior under dark conditions. In contrast, the motion of Fe_2_O_3_-Pt and Fe_2_O_3_-Pt-TiO_2_ was significantly enhanced under light irradiation (Videos S1 and S2). Due to the small size of the nanomotors, it is impossible to distinguish their motion orientation. Based on previous reports, we propose that the propulsion of Fe_2_O_3_-Pt-TiO_2_ nanomotors under visible light follows a self-electrophoretic mechanism, characteristic of metal–semiconductor heterostructures, as illustrated in the conceptual scheme in Figure 2c.^16,17,39^ The MSD plots (Figures 2d–2g) confirm that the motion dynamics of the α-Fe_2_O_3_-based nanomotors are of the diffusion type. Remarkably, the Fe_2_O_3_-Pt-TiO_2_ displays the steepest MSD slope, indicating the highest effective diffusion coefficient (D_e_ = MSD/(4Δt)), which is attributed to its unique heterojunction structure formed between α-Fe_2_O_3_, Pt and TiO_2_. It is worth mentioning that such heterojunction (n-n type) is also theoretically formed in the Fe_2_O_3_-TiO_2_ sample, however, its motion efficiency under light irradiation remained negligible. This observation prompted a closer investigation into the role of the Pt interlayer in facilitating charge transfer and enhancing photocatalytic propulsion in Fe_2_O_3_-Pt-TiO_2_ nanomotors.

**Figure 2:**
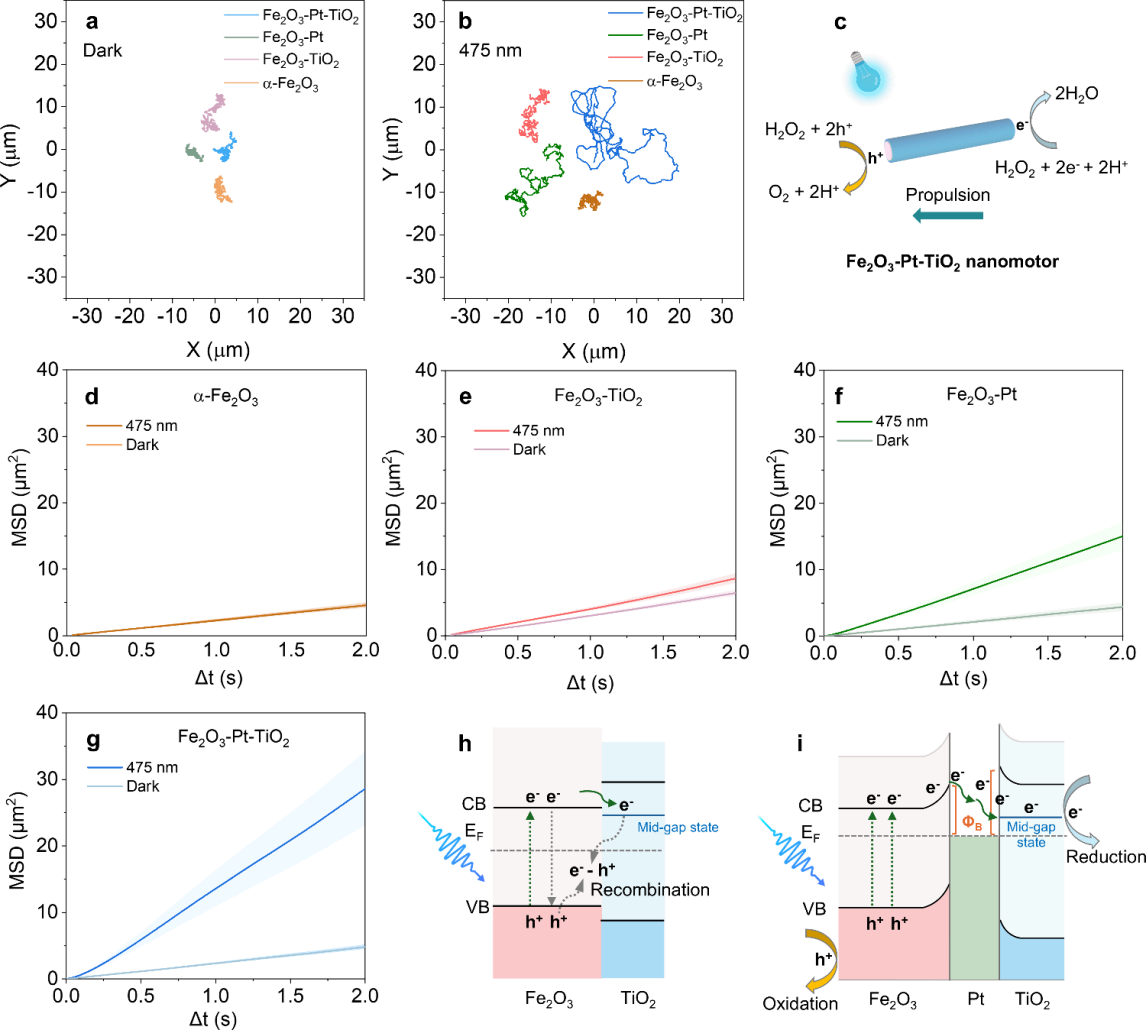
Motion characterization of α-Fe_2_O_3_-based nanomotors. Representative tracking trajectories of the as-synthesized nanomotors recorded over 20 s under dark (a) and 475 nm light irradiation (b). (c) Schematic illustration of the proposed propulsion mechanism of Fe_2_O_3_-Pt-TiO_2_ nanomotors in H_2_O_2_ under 475 nm blue light. (d–g) Plots of averaged MSD versus Δt analyzed from tracking trajectories of α-Fe_2_O_3_, Fe_2_O_3_-TiO_2_, Fe_2_O_3_-Pt, and Fe_2_O_3_-Pt-TiO_2_ nanomotors. Results are shown as the mean ± standard error of the mean, *N* = 20 nanomotors. (h) and (i) Schematic illustration of charge-transfer processes under blue light in different α-Fe_2_O_3_-based heterostructured nanomotors. Φ_B_ represents the Schottky barrier height. Since the work functions of metal and semiconductors were not measured in this work, the mechanism is proposed based on previous literature.^36–38^

Most previous studies on Fe_2_O_3_-TiO_2_-based micromotors rely on UV light to activate both semiconductors. However, under visible light, where only α-Fe_2_O_3_ is active, this heterojunction has shown negligible contribution to propulsion and remains poorly understood.^21^ Consistent with a previous study,^21^ the motion of Fe_2_O_3_-TiO_2_ in this work did not show noticeable enhancement under visible light (Figure 2e). This limited performance may be due to the fact that, although a small amount of photoexcited charges can transfer from α-Fe_2_O_3_ to adjacent TiO_2_, the low carrier mobility and short hole diffusion length (ca. 2–4 nm) result in rapid electron–hole recombination (Figure 2h).^32^ As a result, the ability of Fe_2_O_3_-TiO_2_ to generate propulsive force is significantly hindered. In contrast, the Fe_2_O_3_-Pt-TiO_2_ nanomotors contain a Pt interlayer that acts as a metallic mediator, promoting efficient charge separation and transport between the α-Fe_2_O_3_ and TiO_2_ components.

In principle, the different work functions of Pt (4.5 eV), α-Fe_2_O_3_ (5.5 eV) and TiO_2_ (5.3 eV),^40,41^ lead to the formation of Schottky barriers at the Fe_2_O_3_/Pt and TiO_2_/Pt interfaces under equilibrium conditions, as illustrated in Figures 2i and S2a. Upward band bending at a Schottky contact typically creates a potential barrier that inhibits electron migration from the semiconductor to the metal. However, under blue illumination, a fraction of electrons can acquire enough energy (thermal or kinetic energy) to overcome the barrier toward Pt. Moreover, the Schottky barrier, in turn, could play a beneficial role in suppressing electron backflow from Pt to α-Fe_2_O_3_, thereby reducing recombination and indirectly enhancing charge separation. These electrons accumulated at the Pt interface may then transfer into mid-gap states in TiO_2_ before being injected into the liquid interface.^42^ Additionally, due to the heterogeneous surface and asymmetric shape of nanomotors, the generated photocatalytic species would be unevenly distributed, contributing to their motion behavior.^43^

For the Fe_2_O_3_-Pt sample (Figure 2f), light illumination results in enhanced diffusive motion, consistent with previous findings on noble metal-capped micro/nanomotors. Nevertheless, it is important to note that the diffusion coefficient is still lower than that of Fe_2_O_3_-Pt-TiO_2_, likely due to faster electron–hole pair recombination, which reduces the number of electrons available to generate reactive species (Figure S2b). It is well-known that the rate of a photocatalytic reaction depends on the number of charge carriers that successfully reach the surface. However, charge recombination often competes with surface reactions, occurring on a much faster time scale (10^–9^ s versus 10^–8^–10^–3^ s).^44^ As a result, although some electrons can transfer to the adjacent Pt layer, the short diffusion length leads to partial recombination, reducing propulsion efficiency. Additionally, the exposed Pt surface may directly catalyze H_2_O_2_ decomposition, triggering competing reactions that could act in opposite directions and further limit the motion of the nanomotors.

To further evaluate the role of the heterojunction architecture, we tested the motion behavior of various α-Fe_2_O_3_-based control nanomotors with systematically modified structures: (1) an insulating end layer (Fe_2_O_3_-Pt-SiO_2_), (2) thermally enhanced TiO_2_ crystallinity (Fe_2_O_3_-Pt-TiO_2_-Cal and Fe_2_O_3_-TiO_2_-Cal; Cal denotes calcination), and (3) altered Pt/TiO_2_ positioning (Fe_2_O_3_-TiO_2_-Pt). None of these configurations showed a significant improvement in propulsion under light irradiation. In particular, the insulating SiO_2_ layer in Fe_2_O_3_-Pt-SiO_2_ hindered electron transfer from Pt to the outer surface (Figure S2c), promoting charge recombination and limiting photocatalytic reactions. In the case of Fe_2_O_3_-Pt-TiO_2_-Cal and Fe_2_O_3_-TiO_2_-Cal samples (Figure S3), the annealing process could increase the crystallinity of TiO_2_ and eliminate the internal bands/defects, which in fact reduced the electron trapping ability of TiO_2_.^45^

To gain deeper insight into the enhanced photocatalytic activity observed in Fe_2_O_3_-Pt-TiO_2_, we first performed the steady-state photoluminescence (PL) and time-resolved PL spectroscopy on all α-Fe_2_O_3_-based nanomaterials. The steady-state PL spectra (Figure S4) show that the Fe_2_O_3_-Pt-TiO_2_ heterojunction exhibits the lowest emission intensity, indicating the most efficient suppression of electron–hole recombination among all samples. Moreover, the slowest decay profile in Figure 3a confirms that Fe_2_O_3_-Pt-TiO_2_ exhibits the longest excited carrier lifetime compared to that of other samples.^46,47^ In addition, the electrochemical impedance spectra (EIS, Nyquist plots) under blue light irradiation were collected to investigate the intrinsic photoelectrochemical properties of α-Fe_2_O_3_-based nanomotors (Figure 3b). Evidently, the Fe_2_O_3_-Pt and Fe_2_O_3_-Pt-TiO_2_ structures resulted in a great reduction of charge transfer resistance, wherein the Fe_2_O_3_-Pt-TiO_2_ exhibited the smallest arc radius, which implies the lowest electron transport resistance and therefore the highest charge separation efficiency. Moreover, the photocurrent responses (Figure 3c) demonstrate that the presence of the Pt layer greatly enhances charge carrier transfer between α-Fe_2_O_3_ and TiO_2_, well in line with the observations from EIS analysis. These results further validate our hypothesis that the Fe_2_O_3_-Pt-TiO_2_ nanomotor exhibits superior photocatalytic performance owing to its unique metal-assisted heterojunction structure.

**Figure 3:**
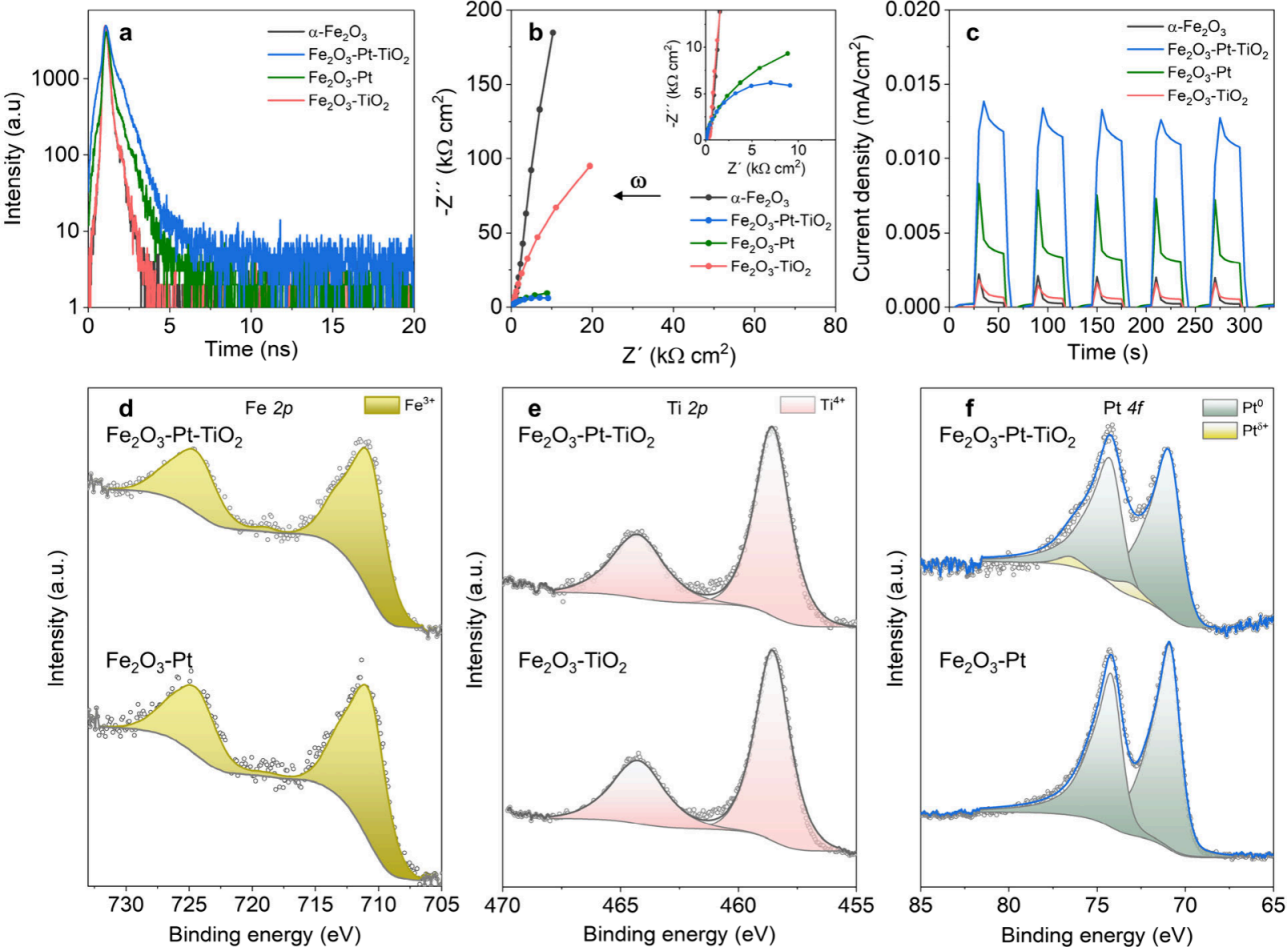
Charge carrier dynamics and surface chemical states of α-Fe_2_O_3_-based nanomotors under light irradiation. (a) Time-resolved photoluminescence decay profiles, λ_em_ = 420 nm, (b) Nyquist plots and (c) transient photocurrent response of α-Fe_2_O_3_, Fe_2_O_3_-Pt-TiO_2_, Fe_2_O_3_-TiO_2_, and Fe_2_O_3_-Pt nanomotors under blue light irradiation, λ = 460 nm. XPS Fe 2p (d), Ti 2p (e), and Pt 4f (f) spectra of Fe_2_O_3_-Pt-TiO_2_, Fe_2_O_3_-TiO_2_, and Fe_2_O_3_-Pt samples using 1600 eV photons and conducted under white light illumination (100 mW/cm^2^) at 3.1 mbar of water pressure.

*In-situ* NAP-XPS analysis was performed to investigate the charge transfer mechanisms and oxidation states of Fe_2_O_3_-Pt-TiO_2_ nanomotors under dark and illuminated conditions. Fe 2p XPS spectra and their fitted results (Figures 3d under illumination and S5a in the dark) show two main peaks located at 710.7 and 724.3 eV in all samples, corresponding to the Fe 2p_3/2_ and 2p_1/2_ orbital peaks of α-Fe_2_O_3_, respectively,^48,49^ providing robust evidence of the formation of α-Fe_2_O_3_ in all measured nanomaterials. In the Ti 2p region (Figures 3e under illumination and S5b in the dark), two different peaks centered at 458.5 and 464.2 eV are observed in all samples, corresponding to the 2p_3/2_ and 2p_1/2_ of Ti^4+^ state of n-type TiO_2_, respectively.^50–52^ Pt 4f XPS data are illustrated in Figures 3f (under illumination) and S5c (in the dark). The results reveal that in Fe_2_O_3_-Pt-TiO_2_, Pt is predominantly present in metallic state (Pt^0^ at 71.0 eV), along with a small fraction of oxidized Pt species (denoted as Pt^δ+^). In contrast, only metallic Pt^0^ was detected in the Fe_2_O_3_-Pt samples. Note that the Pt deposition procedure was conducted in an argon atmosphere, without the involvement of oxygen. Hence, we suggest that the small amount of oxidized Pt in Fe_2_O_3_-Pt-TiO_2_ might be due to the strong electronic interaction formed between Pt/Fe_2_O_3_ and Pt/TiO_2_ interfaces. More interestingly, we found that upon light irradiation, the metallic Pt species in Fe_2_O_3_-Pt-TiO_2_ get slightly increased compared with the dark condition, from 88.6% to 93.2%, as shown in Figure S6, implying that the Pt species received electrons during the photocatalytic reaction process. This is direct evidence of the electron transfer that occurs in heterostructured nanomotors upon light illumination. It is worth noting that since the white light LED source used in the *in situ* XPS measurements was not optimized, only a slight increase could be detected. In contrast, for the Fe_2_O_3_-Pt sample, no difference was observed from the Pt XP spectra in the absence and presence of light irradiation. Taken together, from the *in situ* NAP-XPS analysis, we can conclude that in the Fe_2_O_3_-Pt-TiO_2_ configuration, the Pt layer plays an essential role in promoting electron transfer mediated by Schottky barriers at the interface between α-Fe_2_O_3_ and TiO_2_ semiconductors, which in turn leads to enhanced photocatalytic motion performance. Whereas this effect was not observed in the Fe_2_O_3_-Pt and Fe_2_O_3_-TiO_2_ samples, consistent with the motion behavior previously shown in Figure 2.

Furthermore, in order to probe nonemissive states in photoexcited α-Fe_2_O_3_-based heterojunction structured nanomotors, transient absorption spectroscopy (TAS) was employed to study the charge-transfer dynamics at heterojunction interfaces over femtosecond to nanosecond time scales. As depicted in Figures 4a and 4b, upon photoexcitation, three major TAS regions were observed in the visible range, where a negative ΔA signal peaking at ca. 550 nm (area I) was mainly attributed to ground-state bleach (GSB). The positive TAS signal can be divided into two regions: (II) a characteristic positive excited-state absorption (ESAI) band centered at ca. 600 nm, and (III) a broad tail ESAII extending into the near-IR region. Further, the TAS spectral evolution was compared for α-Fe_2_O_3_-based photocatalytic nanomotors (Figure 4c), and the results demonstrated that the fs-TAS signals of the heterojunction systems primarily originated from the α-Fe_2_O_3_ component, as no discernible spectral features were observed when α-Fe_2_O_3_ was coupled with TiO_2_ and/or Pt. Notably, a relative reduction in the intensity of the strong ESAI peak at approximately 600 nm was observed in Fe_2_O_3_-TiO_2_, Fe_2_O_3_-Pt, and Fe_2_O_3_-Pt-TiO_2_ compared to that of bare α-Fe_2_O_3_, suggesting enhanced interfacial charge transfer facilitated by the strong electron coupling between α-Fe_2_O_3_ and TiO_2_/Pt. This is in agreement with the chemical quenching and spectral analysis results of the α-Fe_2_O_3_ suspension in the presence of Ag^+^ as an electron scavenger (see the discussion in SI and Figures S7–S10). Figures 4d and 4e show that no significant kinetic changes are observed at 550 nm; however, at 600 nm, a clear heterojunction-induced effect is evident in the α-Fe_2_O_3_-based samples, characterized by a buildup of the TAS signal reaching its peak within 100 ps. Considering that the polaron formation in metal oxides typically occurs on faster time scales (∼1 ps), the observed rise kinetics are most likely attributed to the accumulation of photogenerated holes within the α-Fe_2_O_3_ layer as a result of rapid electron extraction toward Pt and TiO_2_, particularly in the case of Pt.^53–55^ However, it is of interest to note that while the presence of Pt can effectively enhance interfacial charge carrier separation and promote hole accumulation, as discussed in XPS analysis, this accumulation may lead to undesirable reverse recombination of charge carriers, thereby reducing the overall photocatalytic activity.^56,57^ On the contrary, by coupling TiO_2_, which acts as an electron acceptor and active catalytic surface to suppress the recombination of electron–hole pairs, a more effective transfer of photogenerated carriers can be achieved (Fe_2_O_3_-Pt-TiO_2_). Indeed, following a similar early time TAS signal buildup for Fe_2_O_3_-Pt-TiO_2_ compared to Fe_2_O_3_-Pt, the decay kinetics decelerate after coupling with TiO_2_, likely due to the suppression of electron–hole recombination.^46,58^ However, it should also be noted that, based on the data available, we cannot exclude contributions to the TAS signal from thermal-induced lattice expansion, which may obscure the dynamics of charge carriers.^59–61^ Based on the above analyses, the enhanced motion performance of Fe_2_O_3_-Pt-TiO_2_ with a Pt interlayer can be attributed to the effective suppression of charge carrier recombination, which is well supported by motion tracking, photoelectrochemical measurements and XPS analysis.

**Figure 4:**
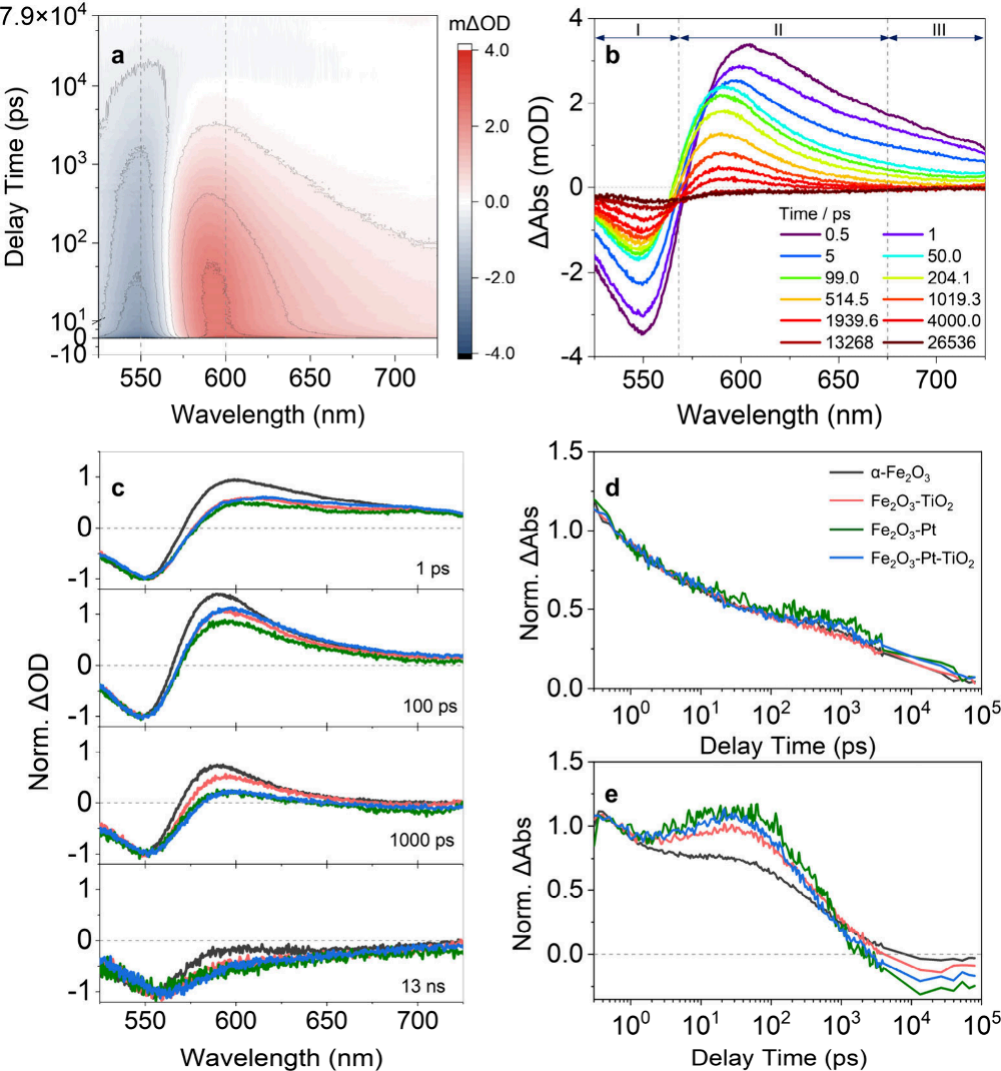
Transient absorption spectroscopy (TAS) analysis of α-Fe_2_O_3_-based nanomotors. (a) 2D TAS contour map of α-Fe_2_O_3_ upon photoexcitation at 500 nm. (b) TAS spectra of α-Fe_2_O_3_ plotted at selected pump–probe delay times. (c) Comparison of TAS spectra normalized to -1 at the negative maximum of bare α-Fe_2_O_3_ and α-Fe_2_O_3_-based heterojunction nanomotors at different delay times and their corresponding TAS kinetics monitored at (d) 550 and (e) 600 nm. A pump wavelength of 500 nm was employed to selectively excite the α-Fe_2_O_3_ support.

Furthermore, the mechanistic study was verified by using the photocatalytic degradation of organic pollutant MB as a model reaction in the presence of the as-prepared samples. The most intense absorption peaks, located at around 663 nm and a shoulder peak at 613 nm, are associated with an MB monomer and dimer, respectively. As shown in Figure 5, after 2 hours of light irradiation, Fe_2_O_3_-TiO_2_ and Fe_2_O_3_-Pt removed only 25% and 48% of MB, respectively. In contrast, Fe_2_O_3_-Pt-TiO_2_ nanomotors achieved a remarkable 91% MB removal within the same time, after which the degradation rate remains almost unchanged (Figure S11), demonstrating significantly higher photocatalytic efficiency compared to the reference samples. Noteworthy, a very small amount of MB degradation was also observed in the absence of any α-Fe_2_O_3_-based nanomaterials under light irradiation, which is because MB undergoes photolysis in the presence of H_2_O_2_ and decomposes itself under visible light (Figures 5d and S11d).^62^ The MB solution with and without H_2_O_2_ under light irradiation was also tested as control experiments (Figures S11e and S11f), both showing negligible degradation. A reusability test was conducted for the Fe_2_O_3_-Pt-TiO_2_ nanomotors, showing that their photocatalytic activity and structural integrity remained stable after three consecutive cycles (Figures S11h, S11i, S12, S13, and S14).

**Figure 5:**
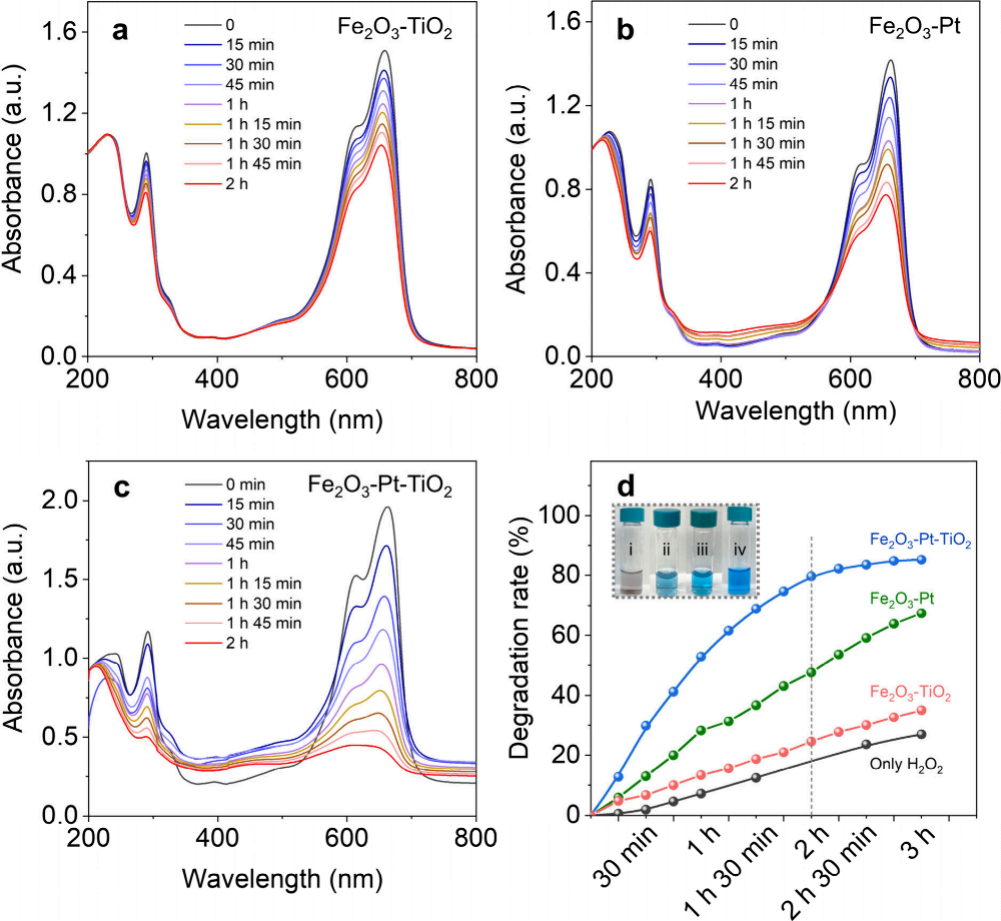
Photocatalytic MB degradation of α-Fe_2_O_3_-based nanomotors. (a–c) Time-dependent UV–visible spectra of methylene blue solution (containing 0.1% H_2_O_2_) in the presence of Fe_2_O_3_-TiO_2_, Fe_2_O_3_-Pt, and Fe_2_O_3_-Pt-TiO_2_ under blue light illumination, respectively. (d) Degradation rate curve for MB solution with and without α-Fe_2_O_3_-based nanomotors. The inset included in (d) is a photograph of MB solution after photodegradation over various samples: (i) Fe_2_O_3_-Pt-TiO_2_, (ii) Fe_2_O_3_-Pt, (iii) Fe_2_O_3_-TiO_2_, and (iv) only H_2_O_2_.

In a typical H_2_O_2_-assisted photocatalytic degradation of MB, the photogenerated carriers react with H_2_O_2_ to produce ^•^OH species, which are highly oxidative and responsible for breaking down MB molecules (Figure S11g).^63–67^ The MB degradation results are consistent with the observed motion performance (Figure 2). A comparison of the photocatalytic efficiency of Fe_2_O_3_-Pt-TiO_2_ nanomotors with previously reported Fe_2_O_3_-based materials is summarized in Table S1, demonstrating equal or superior MB degradation performance despite significantly lower catalyst loading and reduced light intensity. In summary, this study demonstrates that Fe_2_O_3_-Pt-TiO_2_ heterostructured nanomotors exhibit enhanced photocatalytic motion under visible light due to the formation of Schottky barriers mediated by the presence of Pt at the semiconductor-semiconductor interface. This heterojunction, which has been rarely used for nanomotor design, provides unique charge separation, a key factor in achieving improved propulsion. In contrast, Fe_2_O_3_-TiO_2_ nanorods did not show the expected enhancement, as revealed by *in situ* NAP-XPS, TAS, PL, and photoelectrochemical measurements. Therefore, our findings provide fundamental insights into the design of photocatalytic micro/nanomotors, particularly through metal-assisted semiconductor/semiconductor coupling, and emphasize the critical role of mechanistic understanding in refining fabrication strategies for advanced light-driven nanosystems.

## Supplementary Material







## Notes

Experimental methods, FT analysis of HRTEM image, UV–visible spectra of α-Fe_2_O_3_-based nanomaterials, XRD profiles, schematic illustration of band structure of different nanomotors, MSD plots, photoluminescence spectra, XPS under dark condition, atomic ratios of Pt obtained from XPS data, TAS discussion, TAS spectra, time-dependent UV–visible spectra of MB solution over different photocatalytic nanomotors and kinetic fitting results of MB degradation over Fe_2_O_3_-Pt-TiO_2_, characterizations of Fe_2_O_3_-Pt-TiO_2_ after photocatalytic degradation of MB, summary of recently reported Fe_2_O_3_-based photocatalysts for MB degradation under light irradiation, and BET analysis of α-Fe_2_O_3_ nanorods (DOCX)
Video S1, the motion of bare α-Fe_2_O_3_ nanorod under dark and blue light illumination in 0.1% H_2_O_2_ (MP4)
Video S2, the motion of Fe_2_O_3_-Pt-TiO_2_ under dark and blue light illumination in 0.1% H_2_O_2_ (MP4)
