# Supplementary material for: Unraveling Charge Transport in Heterostructured Nanomotors for Efficient Photocatalytic Motion
Source: Nano Lett. 2025 Jun 16;25(25):10169–77. doi: 10.1021/acs.nanolett.5c02177 (PMC12203641; doi:10.1021/acs.nanolett.5c02177)
Supplement: Supplementary file 1 [file nl5c02177_si_001.docx]

**Supplementary Information**

**Unraveling Charge Transport in Heterostructured Nanomotors for Efficient Photocatalytic Motion**

Yufen Chen,^a^ Chunyu Li,^b^ Rebeca Ferrer Campos,^a,c^ María José Esplandiu*,^d^ Jordi Fraxedas, ^d^ Nicoletta Liguori,* ^b^ Katherine Villa* ^a^

^a^ Institute of Chemical Research of Catalonia (ICIQ-CERCA), The Barcelona Institute of Science and Technology (BIST), Tarragona E-43007, Spain

^b^ Institut de Ciències Fotòniques (ICFO), The Barcelona Institute of Science and Technology, Barcelona 08860, Spain

^c^ Departament de Química Física i Inorgànica, Universitat Rovira i Virgili, 43007 Tarragona, Spain

^d^ Catalan Institute of Nanoscience and Nanotechnology (ICN2), CSIC and BIST, Campus UAB, Bellaterra, 08193 Barcelona, Spain

**1. Experimental section**

**1.1 Reagents**

Ferric chloride (FeCl_3_, Sigma-Aldrich 97%), sodium nitrate (NaNO_3_, Cymit Química SL, 99%), fluorine-doped tin oxide glass (FTO, Sigma-Aldrich, surface resistivity ca. 7Ω/sq), absolute ethanol and Milli-Q water were routinely used.

**1.2 The synthesis of α-Fe_2_O_3_ nanorods**

α-Fe_2_O_3_ nanorods were synthesized on fluorine-doped tin oxide (FTO) glass substrate through a one-step hydrothermal process (Fig. 1a).^1^ Prior to use, the FTO substrate was cleaned with water, ethanol and acetone under ultrasound for 10 min, alternatively. 30 mL aqueous solution containing 0.15 M FeCl_3_, and 1 M NaNO_3_ was transferred to a 100 mL Teflon‐lined stainless-steel autoclave. Clean FTO substrates were placed in the Teflon container with the conductive side facing down and heated at 95 °C for 4 hours. As a result, a uniform yellowish layer of iron oxyhydroxide (FeOOH) nanowires was formed on the FTO substrate. Then, the FeOOH‐coated FTO substrate was rinsed with Milli-Q water thoroughly to remove the residual salts, followed by calcination in air at 550 °C for 2 hours with a heating rate of 5 °C min^‐1^. Finally, a reddish α-Fe_2_O_3_ nanowire film was obtained.

**1.3 Synthesis of Fe_2_O_3_-Pt-TiO_2_ nanomotors and other α-Fe_2_O_3_-based samples**

Fe_2_O_3_-Pt-TiO_2_ nanomotors were fabricated by sequential sputtering deposition of 10 nm Pt and ca. 10-15 nm TiO_2_ with an RF-DC magnetron sputtering system. For simplicity, we refer to bare nanorods as α-Fe_2_O_3_, while using Fe_2_O_3_ to denote the heterojunction structures. Importantly, the α-Fe_2_O_3_ phase remained unchanged during the sputtering process. Reference samples, including Fe_2_O_3_-Pt, Fe_2_O_3_-TiO_2_, Fe_2_O_3_-Pt-SiO_2_ and Fe_2_O_3_-TiO_2_-Pt, were prepared using a similar procedure as for Fe_2_O_3_-Pt-TiO_2_. The thicknesses of the deposited Pt, TiO_2_, and SiO_2_ were kept constant at 10 nm, ca. 10-15 nm, and 10-15 nm, respectively. Additionally, the Fe_2_O_3_-Pt-TiO_2_ and Fe_2_O_3_-TiO_2_ samples were calcined at 500 °C for 1 hour (heating rate: 5 °C min⁻¹) and are referred to Fe_2_O_3_-Pt-TiO_2_-Cal and Fe_2_O_3_-TiO_2_-Cal, respectively.

**1.4 Materials Characterization**

Field emission scanning electron microscopy (FESEM) images and energy-dispersive X-ray (EDX) mapping were collected by a focused ion beam scanning electron microscope (Scios 2 by FEI Company). X-ray photoelectron spectroscopy (XPS) experiments were performed at the B07-C ambient pressure (AP)-XPS endstation at the Diamond Light Source synchrotron radiation facility (UK), equipped with a SPECS Phoibos NAP-150 hemispherical electron analyzer using 1600 eV photons and with a pass energy of 40 eV. The experiments were performed under dark and light conditions (Thorlabs MWWHLP2 white light LED, 100 mW/cm^2^) at 3.08 mbar of water pressure. Binding energies are referred to the Pt *4f_7/2_* line of metallic platinum (71.0 eV).^2^ Least-squares fits were performed using the CasaXPS software after subtraction of a Shirley-type background.^3^ The XPS analyses for the samples used in cyclic experiments were performed using a ProvenX-NAP (Specs Surface Nano Analysis GmbH, Berlin, Germany) spectrometer at normal emission with a monochromatic Al Kα X-ray source (100 W, µFocus450) and a spot size of 200 um. Ultraviolet-visible diffuse reflectance spectra were acquired on a UV-vis spectrometer (Shimazu UV-2401PC) with BaSO_4_ as the reference. The absorbance spectrum was obtained by converting the obtained diffuse reflectance data using the Kubelka-Munk function. Bandgap energies (E_g_) were estimated by Tauc plots of [F(R_∞_) E]^2^ vs. photo energy (E), where R_∞_=R_Sample_/R_BaSO4_.

The steady-state photoluminescence (PL) spectra were collected with a Fluorolog Horiba Jobin Yvon spectrofluorometer and an excitation wavelength of 300 nm was used. Time-resolved photoluminescence spectra were carried out on a LifeSpec II spectrometer. Time-correlated single-photon counting mode and a photomultiplier tube (PMT) detector were used for all the measurements. A wavelength of 405 nm EPL picosecond pulsed diode laser was employed as the excitation source. The powder XRD data were obtained using a Bruker AXS D8-Discover diffractometer (40 kV and 40 mA). Grazing incidence X-ray diffraction (GIXRD) was also performed to preferentially detect the thin surface layer of α-Fe_2_O_3_-based nanomaterials on FTO glass substrate. Raman measurements were performed on a confocal Raman spectrometer (Renishaw in Via Qontor). The spectra were gathered with the excitation of 514 nm and 785 nm lasers, with 2400 lines mm^−1^ and 1200 lines mm^−1^ of grating, respectively, in the range of 150–700 cm^−1^ with 1 mW cm^−2^ laser energy. Surface area measurements were conducted on an automatic Micromeritics ASAP 2020 analyzer using N_2_ adsorption isotherms and BET (Brunauer−Emmett−Teller) surface area analysis methods. Samples were degassed under vacuum at 80 °C for 1 h and 110 °C for 20 h before adsorption measurements. The average pore diameter distributions were derived from the desorption branches of the isotherms based on the Barrett-Joyner-Halenda (BJH) model. Electrochemical measurements were carried out on a Bio-Logic VMP3 multichannel potentiostat using a quartz cell (75 mL) with 1 M NaOH (pH 13.6) aqueous solution (30 mL) as the electrolyte. A three-electrode configuration consists of a Pt mesh (ca. 1 cm^2^), an Hg/HgO electrode, and an FTO glass loaded with α-Fe_2_O_3_-based sample as the counter electrode, reference electrode, and working electrode, respectively. Electrochemical impedance spectroscopy (EIS) was performed over a frequency range of 1 MHz to 10 mHz with a 10 mV AC amplitude relative to the open-circuit potential (OCP), under 460 nm LED illumination. Transient photocurrent responses were carried out under on-off cyclic light exposure at 30 s intervals by manually switching on/off the light source at a bias potential of 10 mV (vs. OCP).

The fs-TAS experiments were conducted using a customized dual Yb:KGW regenerative amplified laser system (PHAROS, Light Conversion Ltd., 1030 nm, 250 fs, 400 μJ), operating at a repetition rate of 1 kHz. The setup is described in detail elsewhere.^4^ Approximately 50% of the fundamental 1030 nm beam is utilized to pump an optical parametric amplifier, generating laser pulses centered at 800 and 1450 nm (signal and idler, respectively). The 1450 nm idler beam is directed into a non-collinear optical parametric amplifier (NOPA, ORPHEUS-N-2H, Light Conversion Ltd.), which is also pumped by the PHAROS 1030 nm. The output beam from this NOPA, centered at 500 nm, serves as the pump pulse for sample excitation. The output of an optical parametric amplifier signal generated from another PHAROS and centered at 800 nm is used to generate continuum white light as the probe by focusing the beam onto a YAG crystal. A set of achromatic lenses is used to collimate the continuum, before focusing it onto the sample position (1 mm quartz cuvette), and then refocusing it onto the slit of a prism-based spectrometer. The spectrum is dispersed on a back-thinned FFT-CCD area image sensor (1024 x 64 pixel, Hamamatsu S12600-1006 model). Modulation of the signals is achieved via choppers, with data acquisition on a shot-to-shot basis. The temporal delay between pump and probe pulses is adjustable through optical delay lines (DL-BKIT2U, Newport), allowing for delay times up to 4 ns. For extended pump-probe delays, electronic synchronization of the two PHAROS systems, which comprises two regenerative amplifiers seeded by a shared oscillator, enables delay extension up to nearly 1 ms, with ˜13 ns timesteps, facilitating continuous ΔA tracking. All measurements were run at room temperature. For data analysis, the dataset was preprocessed to correct for the chirp using a Python package KiMoPack.^5^

**1.5 Motion Characterization of α-Fe_2_O_3_-based samples**

The α-Fe_2_O_3_-based nanomotors were scratched off from the FTO glass substrate and dispersed in 0.1 wt% H_2_O_2_ aqueous solution using an ultrasonic bath for 10 minutes. For each test, the nanomotor suspension was placed into a small chamber (9 mm diameter, 0.12 mm depth) on a glass slide and sealed with a coverslip. The motion of the nanomotors was recorded by a THUNDER Imager modular DMi8 inverted microscope from Leica, equipped with Leica Application Suite (LAS) X software. Trajectories were analyzed using a custom Python script to extract tracking data. A 475 nm blue LED integrated into the microscope served as the light source to activate the nanomotors. In each experiment, the motion of 20 nanomotors was recorded for 20 s at 25 fps. The averaged mean-square-displacement (MSD) versus time intervals (∆t) was calculated from the trajectories to study the motion behavior of nanomotors.

**1.6 Photocatalytic performance of α-Fe_2_O_3_-based samples**

The photocatalytic degradation of methylene blue (MB) was performed under a 460 nm blue LED illumination (67 mW/cm^2^). For each measurement, the as-synthesized α-Fe_2_O_3_-based nanowire film (1 cm^2^) was scratched off from the FTO glass and dispersed in 3 mL of MB solution (10 ppm, corresponding to a photocatalyst concentration of 33 mg/L) containing 0.1 wt% aqueous H_2_O_2_. Aliquots were collected every 15 minutes and analyzed using a UV–vis spectrophotometer (Shimadzu UV-1800). Control experiments using MB + H_2_O_2_ and MB aqueous solution were performed to rule out any self-degradation of MB under light illumination. Cyclic experiments were also carried out for Fe_2_O_3_-Pt-TiO_2_ nanomotors. After each cycle, the nanomotors were alternately rinsed with Milli-Q water and ethanol, then dried and recovered for reuse. The degradation rate was calculated by using the equation: degradation rate (%) = [(A_0_-A_t_)/A_0_] *100, where A_0_ and A_t_ refer to the absorbance of MB at time = 0 min and at a specific time.

**2. Supplementary Figures**

Figure S2a shows the non-equilibrium band alignment of Fe_2_O_3_-Pt-TiO_2_, prior to physical contact and without interfacial interactions or band bending. The energies are referred to the Fermi level (E_F_) of the hemispherical analyser described in the manuscript. Numbers in black correspond to the binding energies of the Fe*2p_3/2_*, Pt*4f_7/2_* and Ti*2p_3/2_* core levels (see Fig. 3d-f in manuscript). The 709.5 and 456.0 eV values correspond to the (*E*_Fe2p3/2_ *− E*_VBM_)^Fe2O3^ and (*E*_Ti2p3/2_ *− E*_VBM_)^TiO2^,^6,7^ respectively. Such values are characteristic of the referred materials. Given the known energy values referenced to E_F_ (black numbers), we can determine the corresponding valence band maximum (VBM), namely, 1.5 and 2.8 eV for α-Fe_2_O_3_ and TiO_2_, respectively, and thus determine the band offset (1.3 eV). For metallic Pt, VBM = 0 eV. The (*E*_Ti2p3/2_ *− E*_VBM_)^TiO2^ = 455.8 eV value (in green), corresponds to solution-processed TiO_2_ films (unpublished).

The band diagram is further completed with the positions of the vacuum level (VL). Here, it is important to point out that such position, or equivalently the values corresponding to work function (WF) and ionization potential (IP) are extremely sensitive to the surface conditions (structure, orientation, order/disorder, morphology, coating, contamination, etc.) and this is the reason for the large dispersions found in the literature. In the case of α-Fe_2_O_3_, we select the WF value from Ref. [^8^], which has been determined for single crystals of hematite (α) and determined *in situ*. The 5.5 eV shown in the figure may decrease to approximately 4.5 eV for polycrystalline surfaces that have been exposed to ambient conditions. The 4.5 eV from Pt (in green) has been determined from thin films grown by sputtering on silicon wafers and measured *ex-situ*. In the case of TiO_2_, IP = 8.0 eV stands from a mean value obtained from surfaces that have been exposed to different conditions.^9^ We compare such value to 7.3 and 7.7 eV that were obtained on solution-processed TiO_2_ films.

In summary, the band offset for Fe_2_O_3_-Pt-TiO_2_ can be estimated to be approximately 1.3 eV. The formation of interfacial dipoles at both junctions is anticipated, although accurately determining the energy barrier heights remains challenging. Nonetheless, these barriers are expected to be below 1 eV.


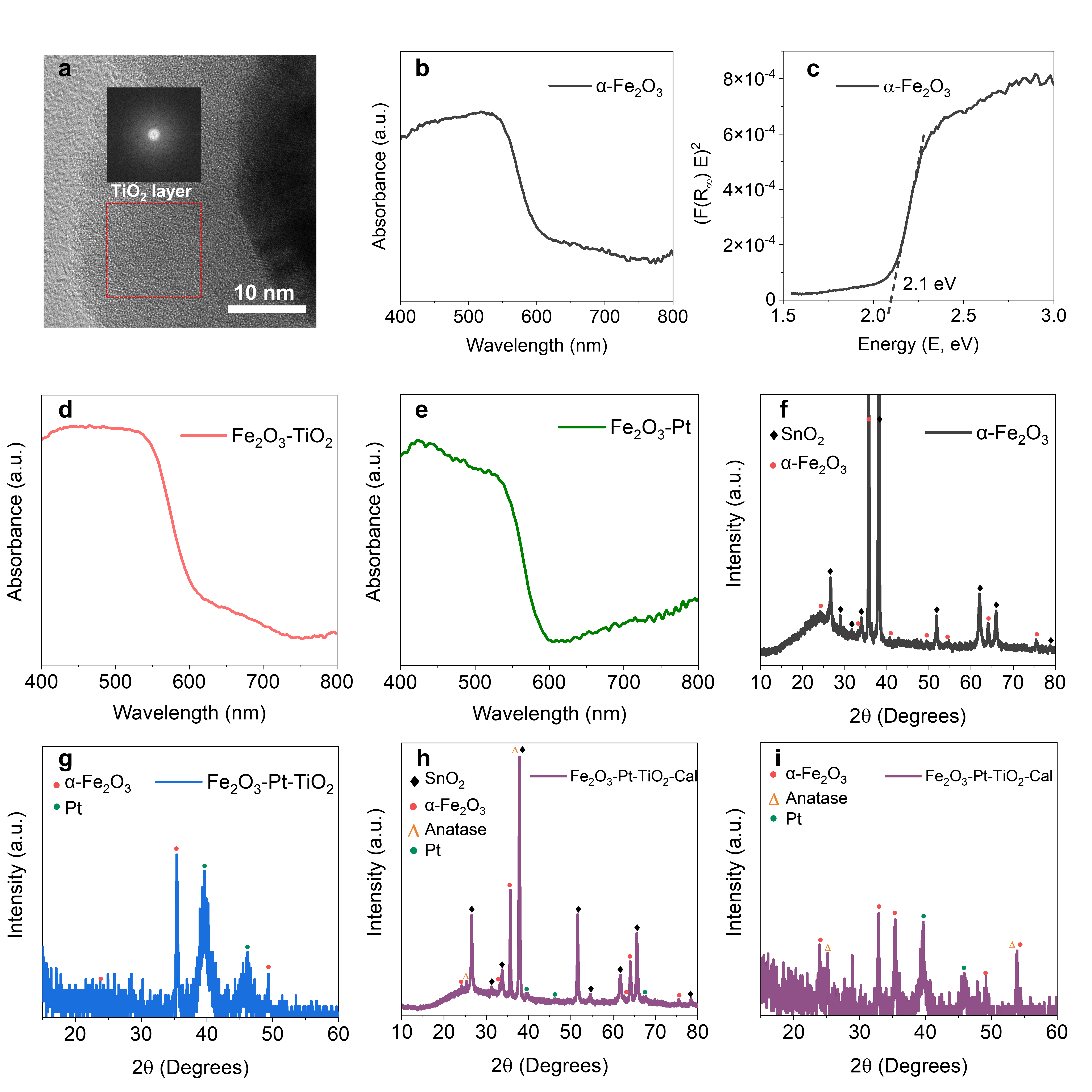


**Figure S1.** Characterizations of α-Fe_2_O_3_-based samples. (a) HRTEM image of Fe_2_O_3_-Pt-TiO_2_. The inset shows the Fourier Transform (FT) image that corresponds to the selected red-framed region in the TiO_2_ layer. The FT analysis indicates that the selected TiO_2_ layer is primarily in an amorphous phase. (b) UV-vis absorption spectra of bare α-Fe_2_O_3_. (c) Tauc plots of (F(R_∞_) E)^2^ versus E(eV) for α-Fe_2_O_3_. The dashed line in (c) is used to determine the bandgap energy. (d) and (e) UV-vis absorption spectra of Fe_2_O_3_-TiO_2_ and Fe_2_O_3_-Pt, respectively. Notably, compared to bare α-Fe_2_O_3_ and Fe_2_O_3_-TiO_2_, both Fe_2_O_3_-Pt and Fe_2_O_3_-Pt-TiO_2_ exhibit slight absorption in the 600–800 nm range. This additional absorption may be attributed to the localized surface plasmon resonance (LSPR) effect of the Pt nanoparticles. The presence of this plasmonic effect could also contribute to the enhanced visible-light response of the Fe_2_O_3_-Pt-TiO_2_ heterostructure. (f) XRD pattern of α-Fe_2_O_3_. (g) GIXRD pattern of Fe_2_O_3_-Pt-TiO_2_, confirming the presence of α-Fe_2_O_3_ (JCPDS No. 01-087-1165) and Pt (JCPDS No. 00-004-0802) phases. (h) and (i) show the XRD and GIXRD patterns of Fe_2_O_3_-Pt-TiO_2_-Cal, respectively. After calcination, both patterns reveal a marked improvement in crystallinity, particularly for the TiO_2_ phase, which corresponds to the anatase structure (JCPDS No. 00-021-1272). This confirms that the TiO_2_ in the as-prepared Fe_2_O_3_-Pt-TiO_2_ sample was initially amorphous.


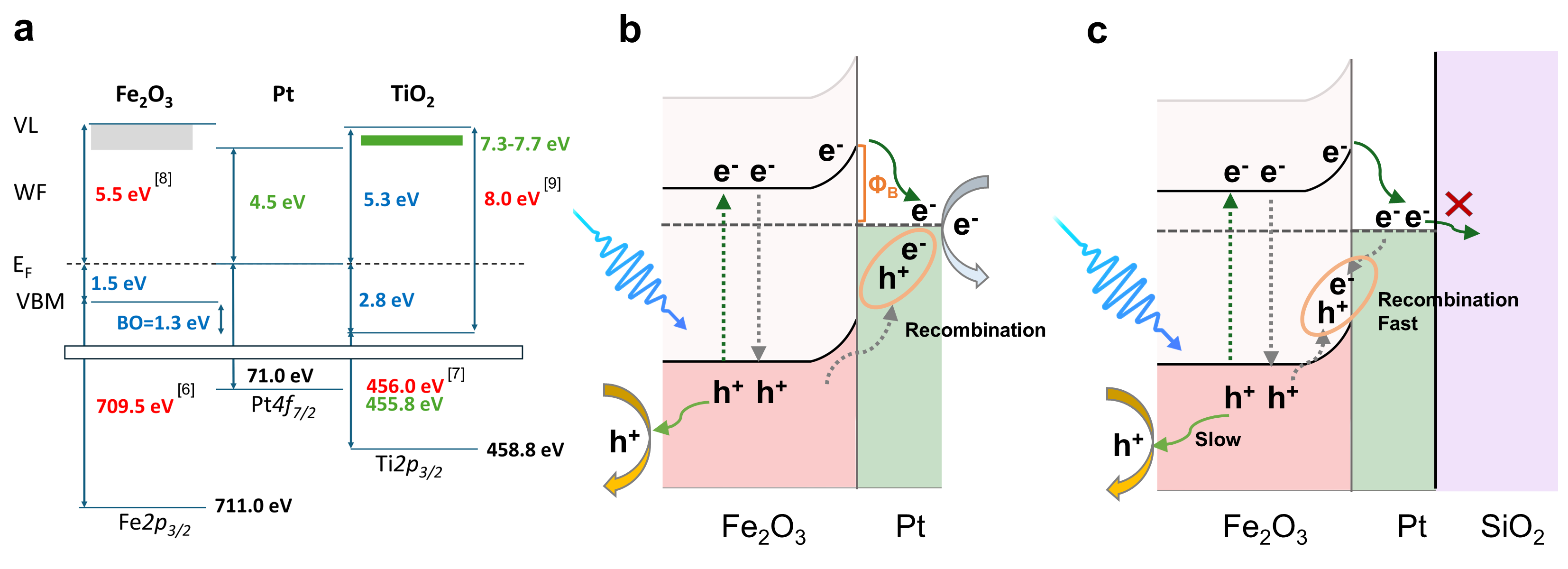


**Figure S2.** Band alignment and charge transfer mechanisms in α-Fe_2_O_3_-based samples. (a) Schematic illustration of the band structure of Fe_2_O_3_-Pt-TiO_2_ nanomotors in non-equilibrium. (b) Charge-transfer processes under blue light in (b) Fe_2_O_3_-Pt and (c) Fe_2_O_3_-Pt-SiO_2_ heterostructures. Φ_B_ represents the Schottky barrier height.


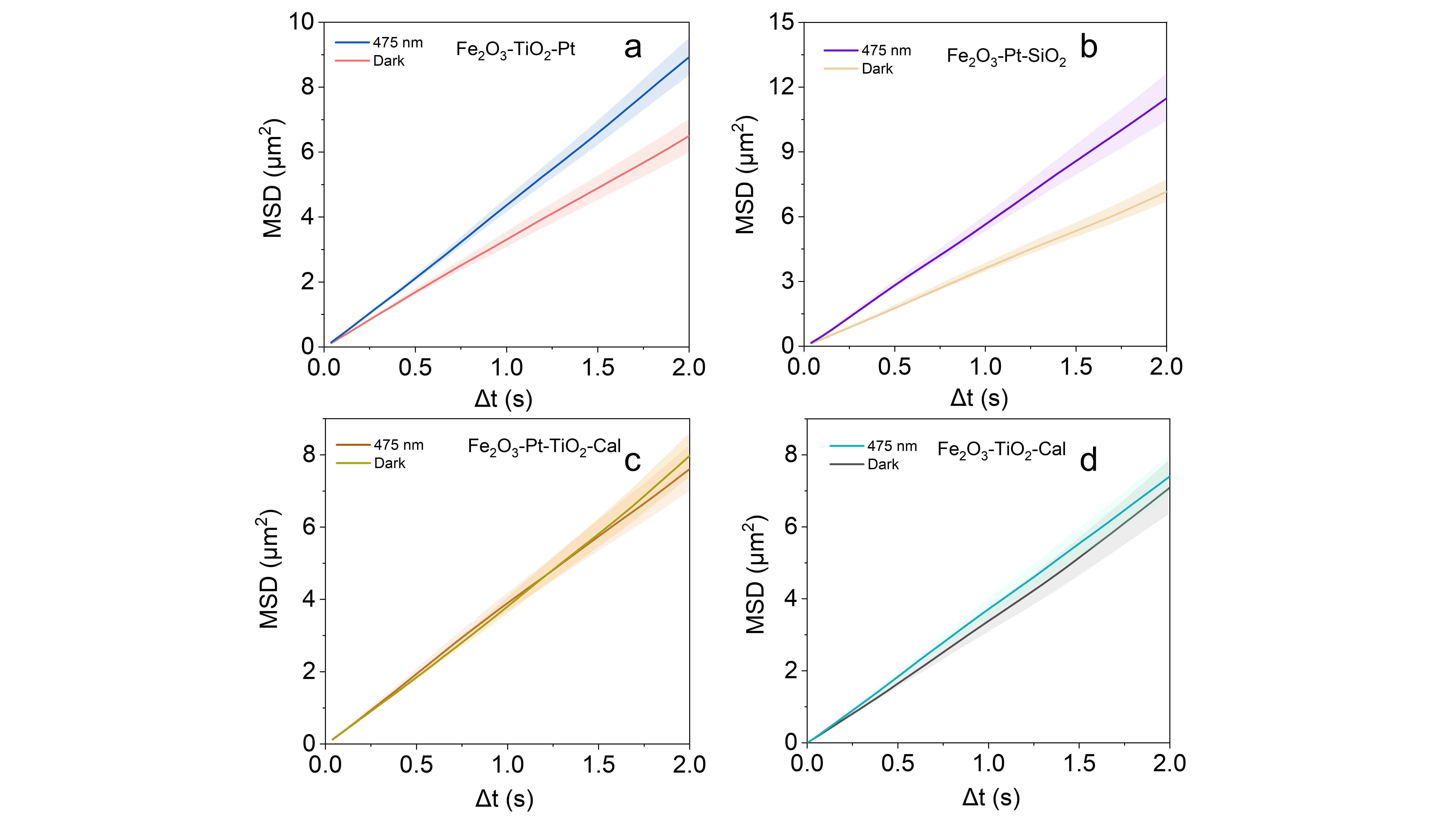


**Figure S3.** Plots of averaged MSD versus ∆t analyzed from tracking trajectories of (a) Fe_2_O_3_-TiO_2_-Pt, (b) Fe_2_O_3_-Pt-SiO_2_, (c) Fe_2_O_3_-Pt-TiO_2_-Cal, and (d) Fe_2_O_3_-TiO_2_-Cal. Results are shown as the mean ± standard error of the mean, N = 20 nanomotors.


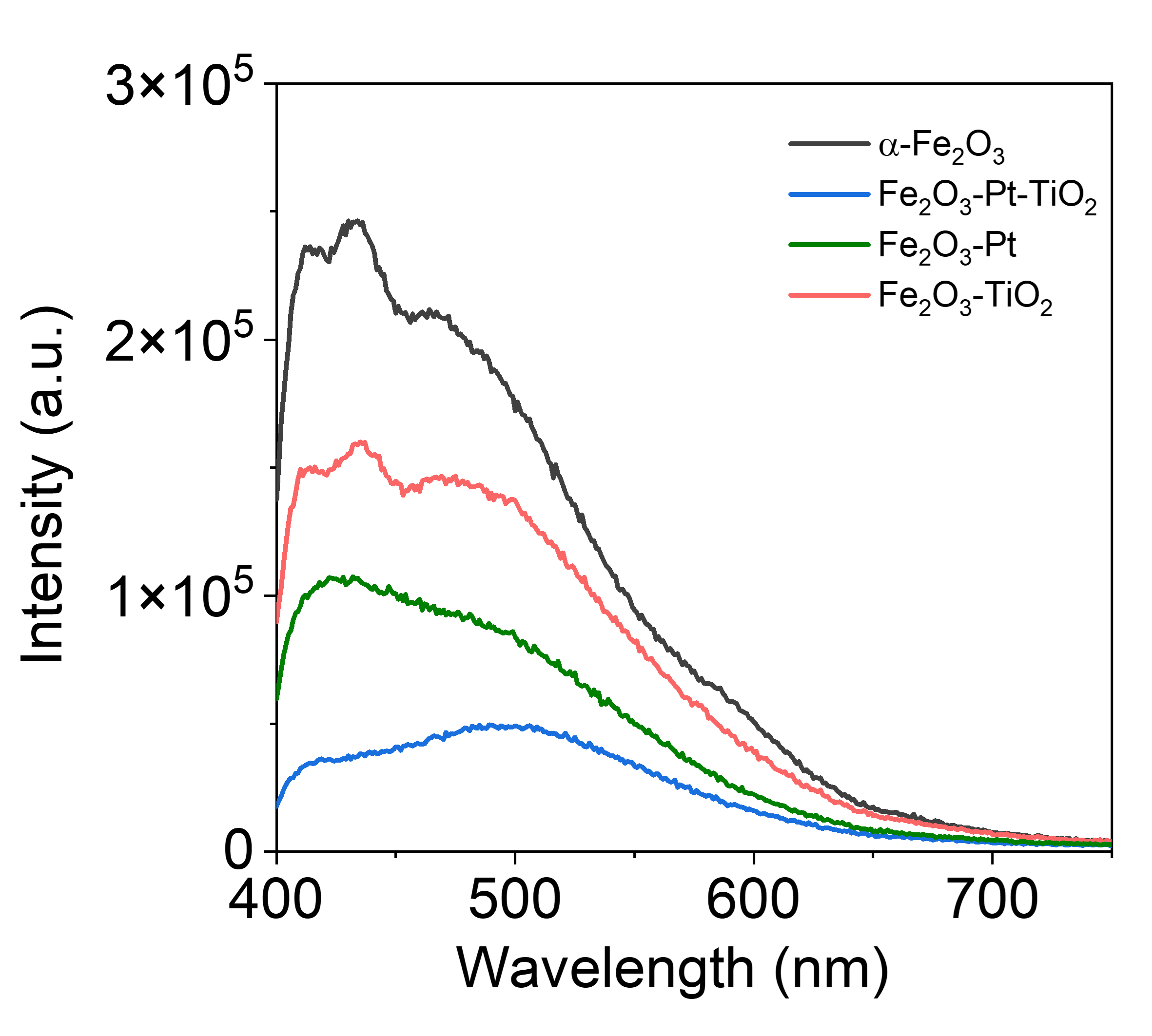


**Figure S4.** Photoluminescence spectra of Fe_2_O_3_-Pt-TiO_2_, Fe_2_O_3_-Pt, and Fe_2_O_3_-TiO_2_ and bare α-Fe_2_O_3_ samples, λ_ex_ = 300 nm.


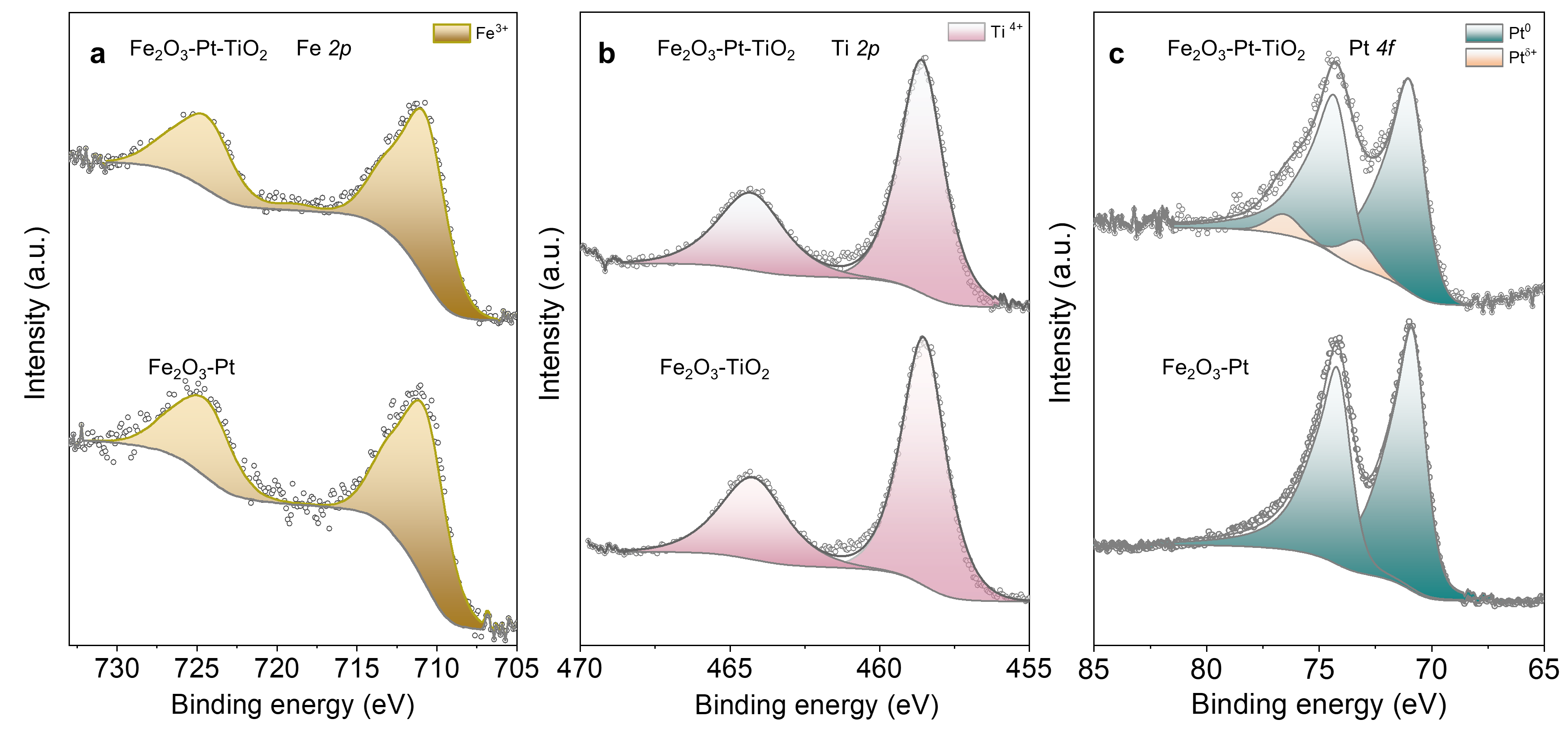


**Figure S5.** XPS Fe 2*p* (a), and Ti 2*p* (b), and Pt 4*f* (c) spectra of Fe_2_O_3_-Pt-TiO_2_, Fe_2_O_3_-Pt, and Fe_2_O_3_-TiO_2_ samples measured under dark conditions using 1600 eV photons at 3.1 mbar of water pressure.


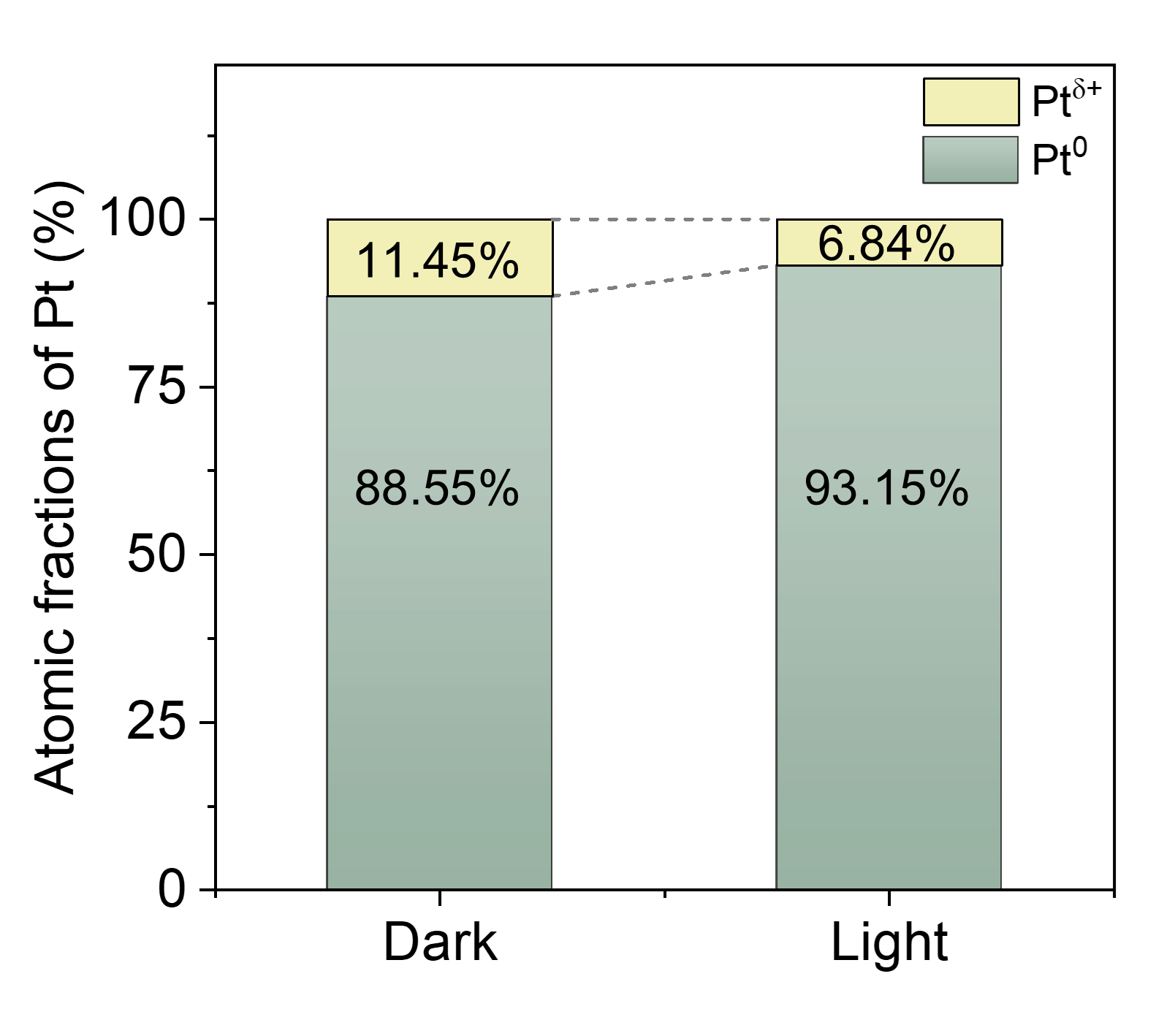


**Figure S6.** The atomic ratios of the different Pt oxidation states extracted from the corresponding XP spectra in Figure 3f and Figure S5c.

**TAS discussion**

To elucidate the nature of excited-state absorption, we compare our results with previous TAS studies of α-Fe_2_O_3_. It has been shown that primarily photogenerated holes give rise to this ESA feature based on the difference in spectra of α-Fe_2_O_3_ thin films at positive bias (“excess” holes).^10–12^ However, alternative assignments of the positive TAS profile after 1 ns to thermal-induced bandgap shrinkage rather than an electronic-state origin have been proposed.^13,14^ To confirm the attribution of TAS signals in our measurements, chemical quenching measurements were carried out for α-Fe_2_O_3_ suspensions in the presence of an electron scavenger (silver nitrate, Ag^+^) and a hole scavenger (methanol, MeOH). As shown in Figure S7a, the presence of Ag⁺ induced an initial decrease in the ESA peak (I), leading to the formation of a broader absorption band spanning the range of 600–725 nm. Consequently, the remaining positive broad absorption feature was primarily attributed to photogenerated holes due to the electron scavenging by the Ag^+^. In contrast, no significant change in the TAS spectra was observed after the addition of MeOH (Figure S7b), implying that photogenerated holes in the α-Fe_2_O_3_ nanorods film appeared to be insensitive to the chemical environment. As can be noted from the TAS spectral evolution within *ca.* 1 ns (Figures 4b and S8a), a blue shift of the ESAI peak was observed, accompanied by the decay of the broad feature from 650 to 725 nm, likely associated with the rapid carrier recombination.^10,15^ In addition, the decay kinetics were found to be highly wavelength-dependent, indicative of the involvement of multiple photoexcited species (Figure S8b).

To confirm the absence of excitation power-dependent dynamics, the TAS experiments for α-Fe_2_O_3_ were performed at various excitation powers, between 340 and 85 nJ per pulse. The TAS kinetics at 550 nm (Figure S9) demonstrated an excitation-power-independent behavior, consistent with a monomolecular first-order process and the absence of multiexciton interactions under the experimental conditions.

Additionally, the Fe_2_O_3_-Pt-SiO_2_ heterojunction system was measured to eliminate potential instrumental artefacts (Figure S10). Only minor spectral or kinetic differences were observed, indicating minimal interfacial effects were generated from SiO_2_ due to its inert nature, well in line with its motion behavior.


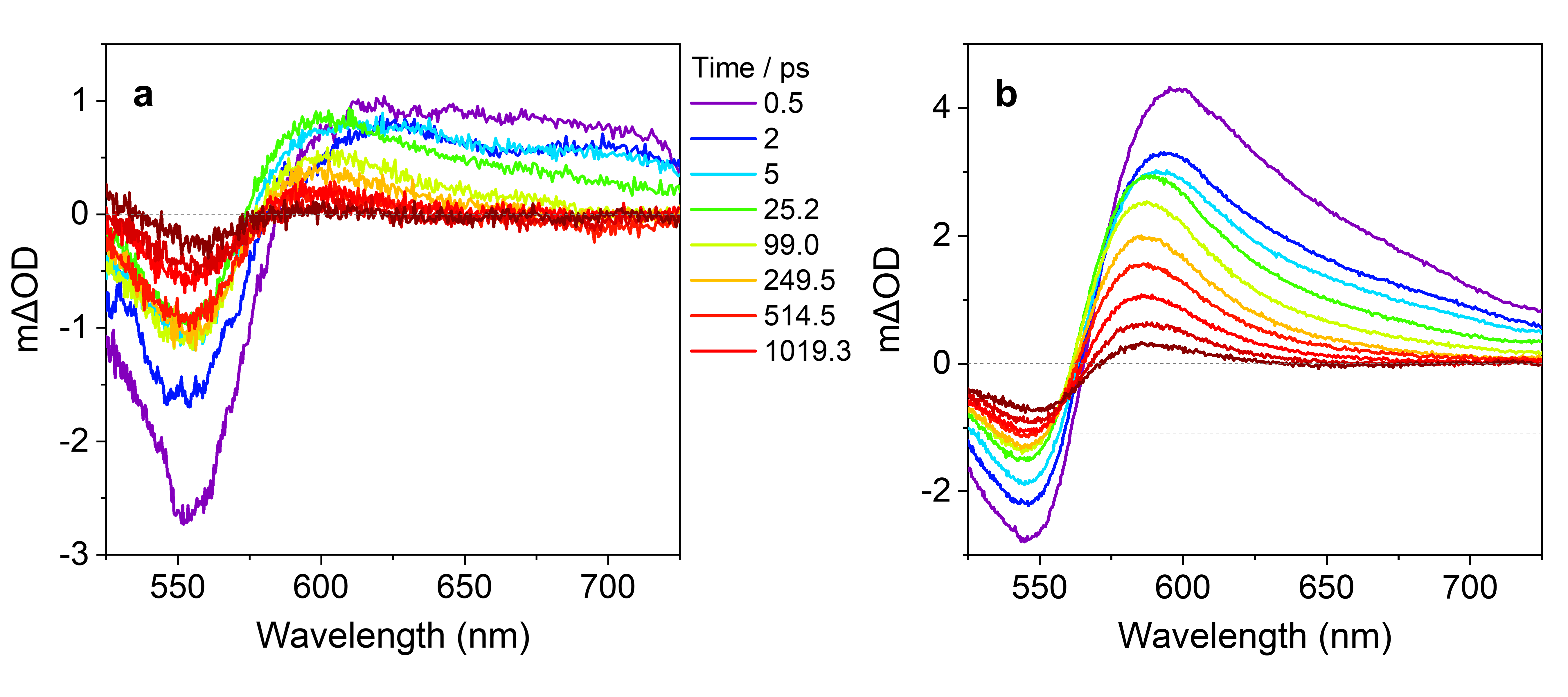


**Figure S7.** TAS spectra of α-Fe_2_O_3_ dispersions in the presence of (a) AgNO_3_ (10 mM) and (b) methanol (10 vol%) excited at 500 nm.


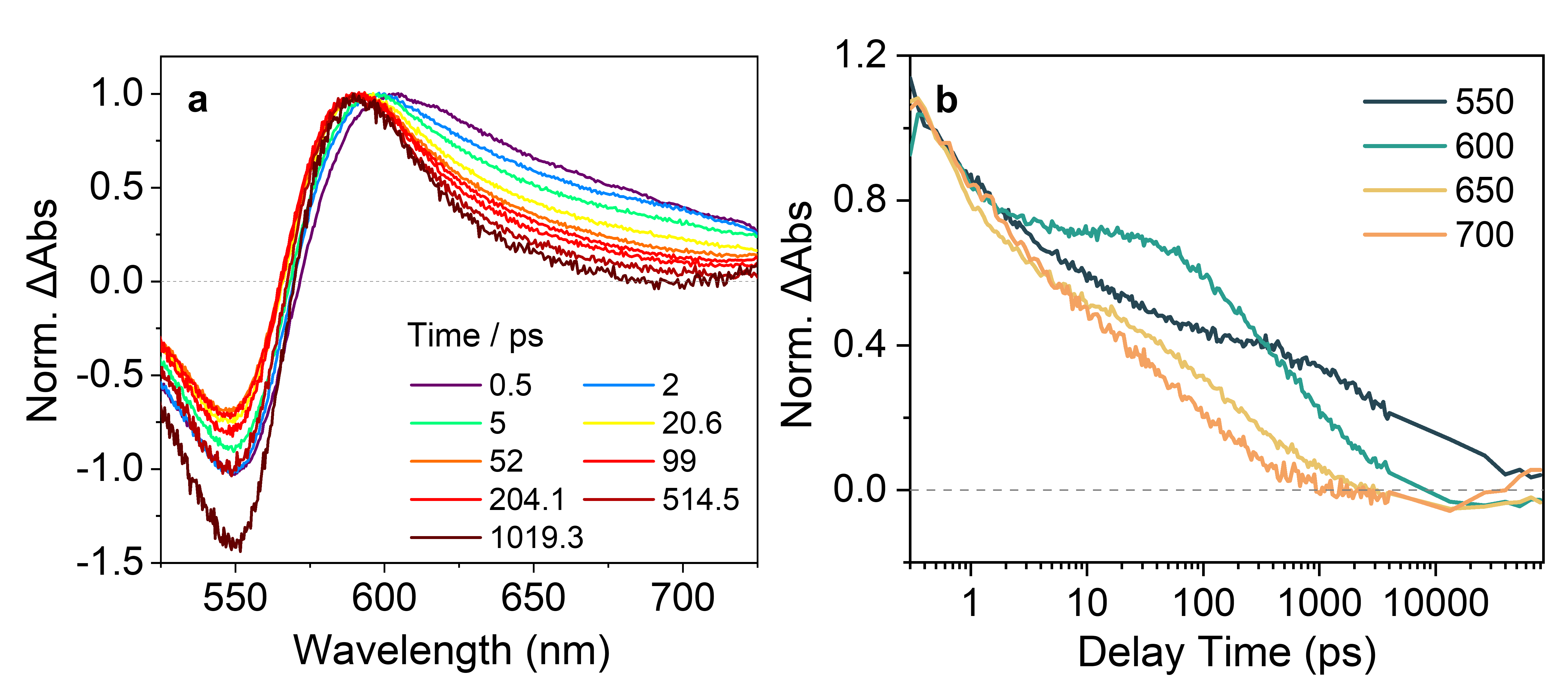


**Figure S8.** (a) TAS spectra of α-Fe_2_O_3_ dispersions normalized at the ESA maximum, and (b) its TAS dynamics monitored at different wavelengths normalized at 0.4 ps.


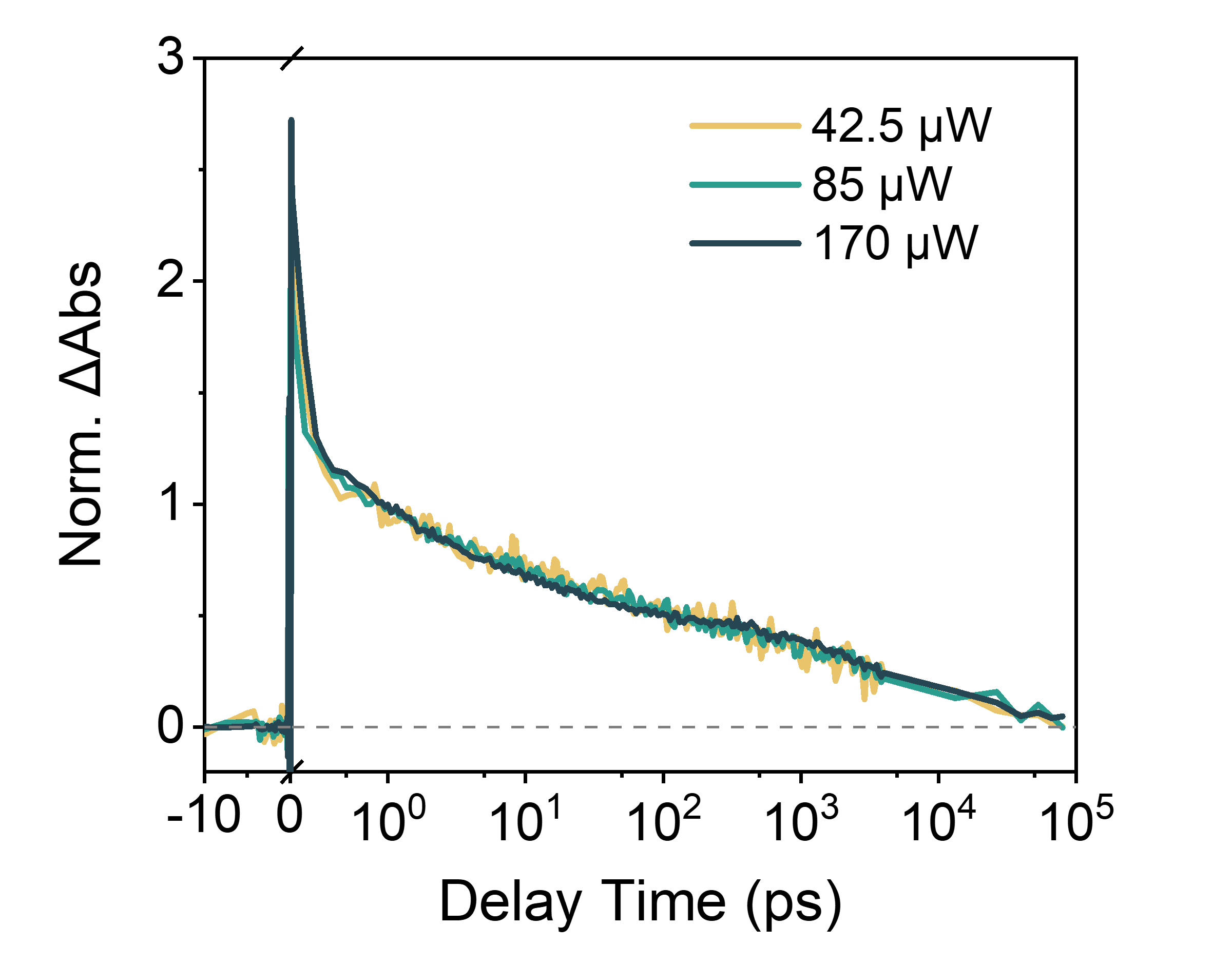


**Figure S9.** fs-TAS decay kinetics of α-Fe_2_O_3_ dispersion probed at 550 nm under varying excitation powers using 500 nm (500 Hz) excitation pulses.


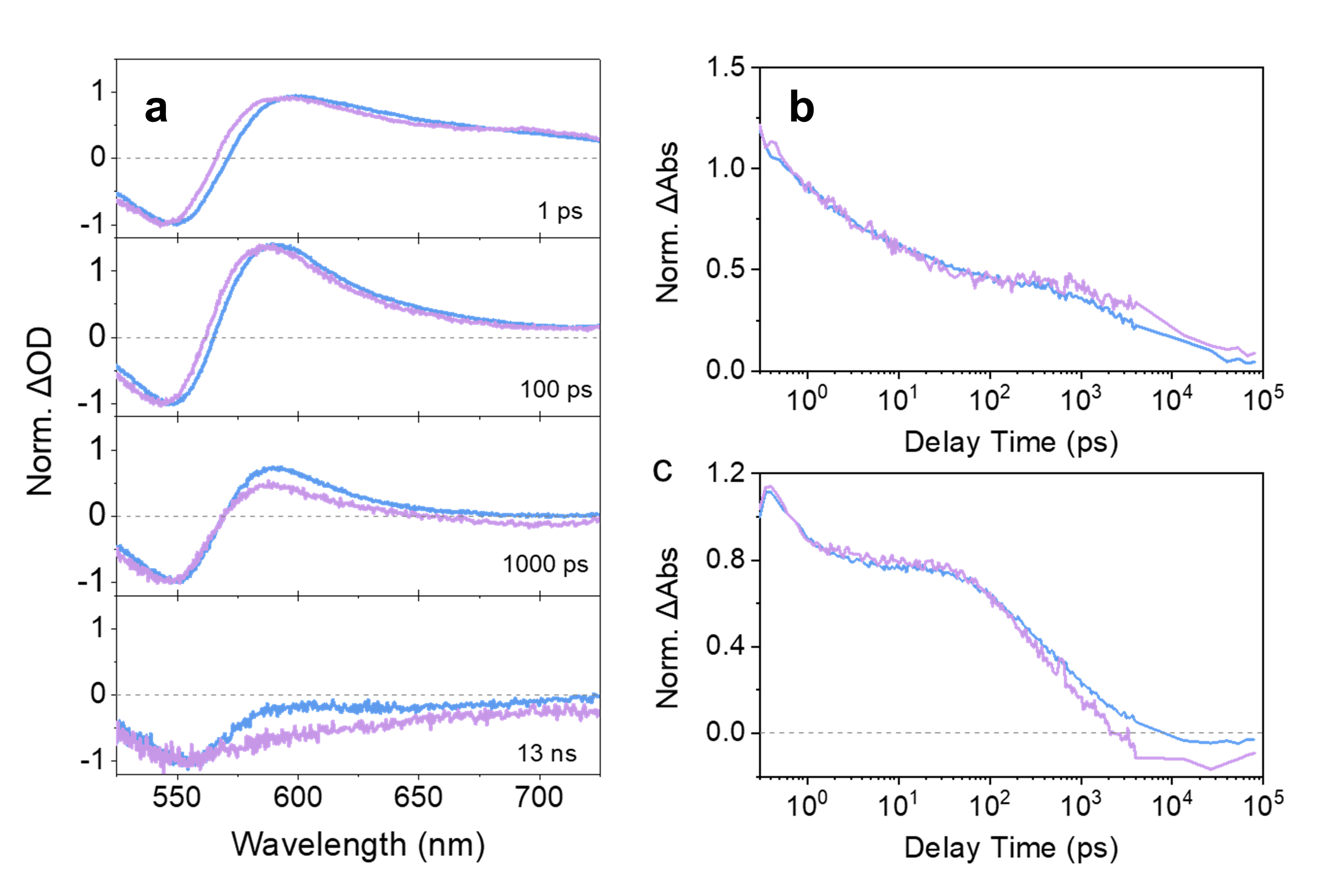


**Figure S10.** (a) TAS spectra recorded at different delay times after 500 nm excitation and normalized with respect to the bleaching maximum for α-Fe_2_O_3_ (blue) and Fe_3_O_3_-Pt-SiO_2_ (pink). The corresponding TAS kinetics probed at (b) 550 nm and (c) 600 nm, respectively.


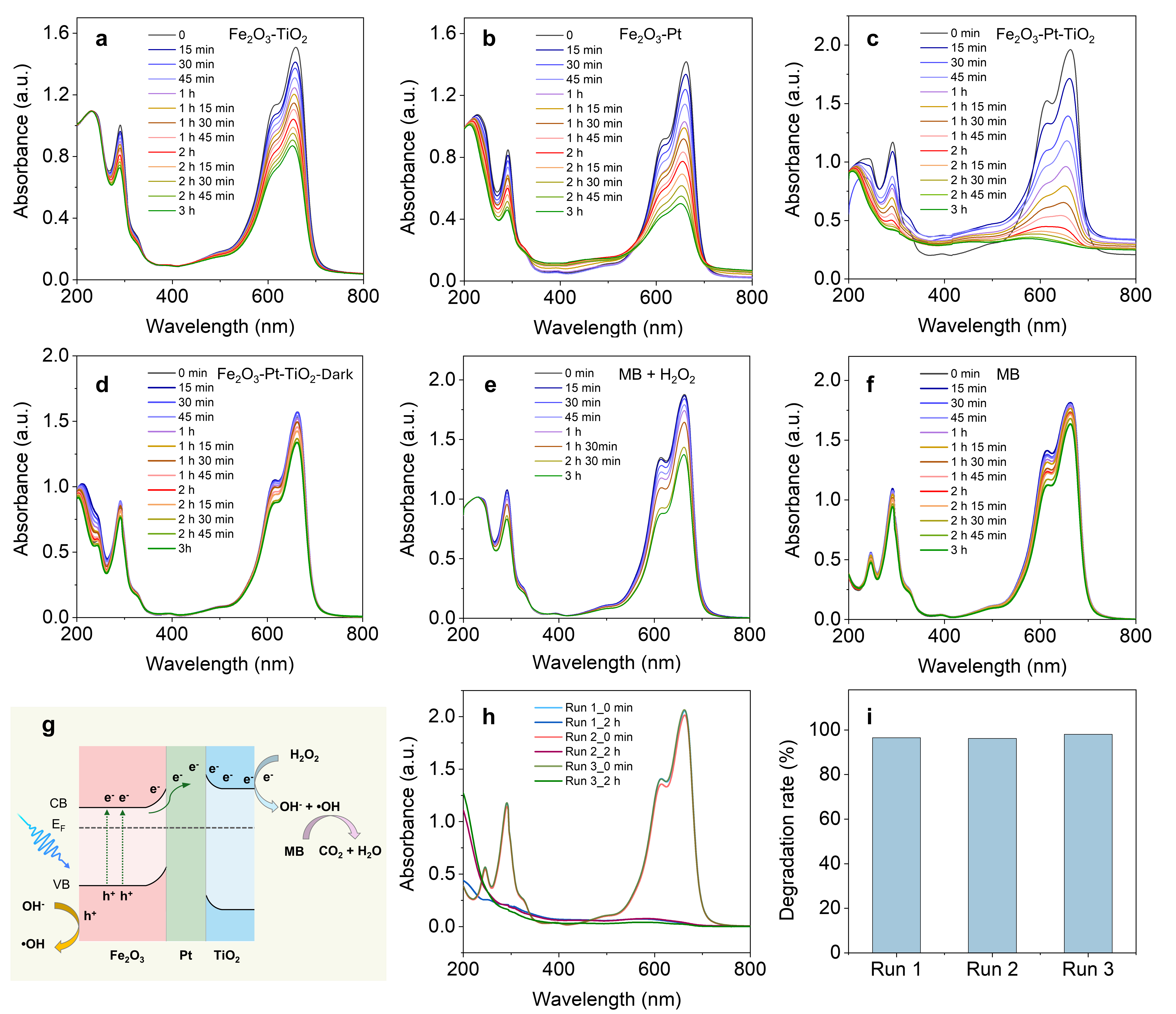
**Figure S11.** Photocatalytic degradation of methylene blue using α-Fe_2_O_3_-based samples under blue light irradiation (a-f) Time-dependent UV-visible absorbance spectra of MB solution (0.1% H_2_O_2_) in the presence of (a) Fe_2_O_3_-TiO_2_, (b) Fe_2_O_3_-Pt, (c) Fe_2_O_3_-Pt-TiO_2_, under light irradiation and (d) Fe_2_O_3_-Pt-TiO_2_ under dark condition, (e, f) control experiments using MB + 0.1 % H_2_O_2_, and MB alone without nanomotors under blue light illumination. (g) Schematic representation of the proposed reaction mechanism of the photocatalytic MB degradation over Fe_2_O_3_-Pt-TiO_2_ nanomotors with H_2_O_2_ under blue light illumination. (h) UV–vis absorbance spectra from cyclic degradation experiments with Fe_2_O_3_-Pt-TiO_2_ nanomotors and(i) Corresponding degradation rates over three consecutive runs, indicating good reusability and photocatalytic stability.

**Photocatalytic MB degradation mechanism.**

Under visible light irradiation, Fe_2_O_3_-Pt-TiO_2_ nanomotor acts as an effective photocatalyst by generating electron-hole pairs (Eq. (1)). Then, the photogenerated h^+^ migrate to the surface and react with water molecules to produce hydroxyl radicals (•OH) (Eq. (2)), which actively oxidize MB. Simultaneously, the photoexcited electrons (e⁻) can reduce Fe^3+^ to Fe^2+^ (Eq. (3)). In the presence of H_2_O_2_, the generated Fe^2+^ undergoes a Fenton reaction with H_2_O_2_ to produce additional •OH radicals (Eq. (4)), thereby further enhancing the degradation of the dye. Hydroxyl radicals (•OH) play a key role in the decomposition of MB during photodegradation (Eq. (5)).

Fe_2_O_3_ + *hv* → *e*^-^ + *h*^+^  (1)

H_2_O + h^+^ → •OH (2)

Fe^3+^ + e^-^ → Fe^2+^  (3)

Fe^2+^ + H_2_O_2_ → Fe^3+^ + OH^−^ + •OH (4)

MB + •OH → CO_2_ + H_2_O (5)

In addition, the reaction kinetics of MB photodegradation within the first 2 hours for different samples can be described by the pseudo-first-order model: ln(C_0_/C) = k_app_t, where k_app_ is the apparent rate constant (min^–1^), C and C_0_ are the concentration after reaction time t and the initial concentration, respectively. As shown in Figure S12, the Fe_2_O_3_-Pt-TiO_2_ nanomotors exhibited the highest rate constant among all synthesized samples, consistent with the photocatalytic performance trends observed in Figure 5d.


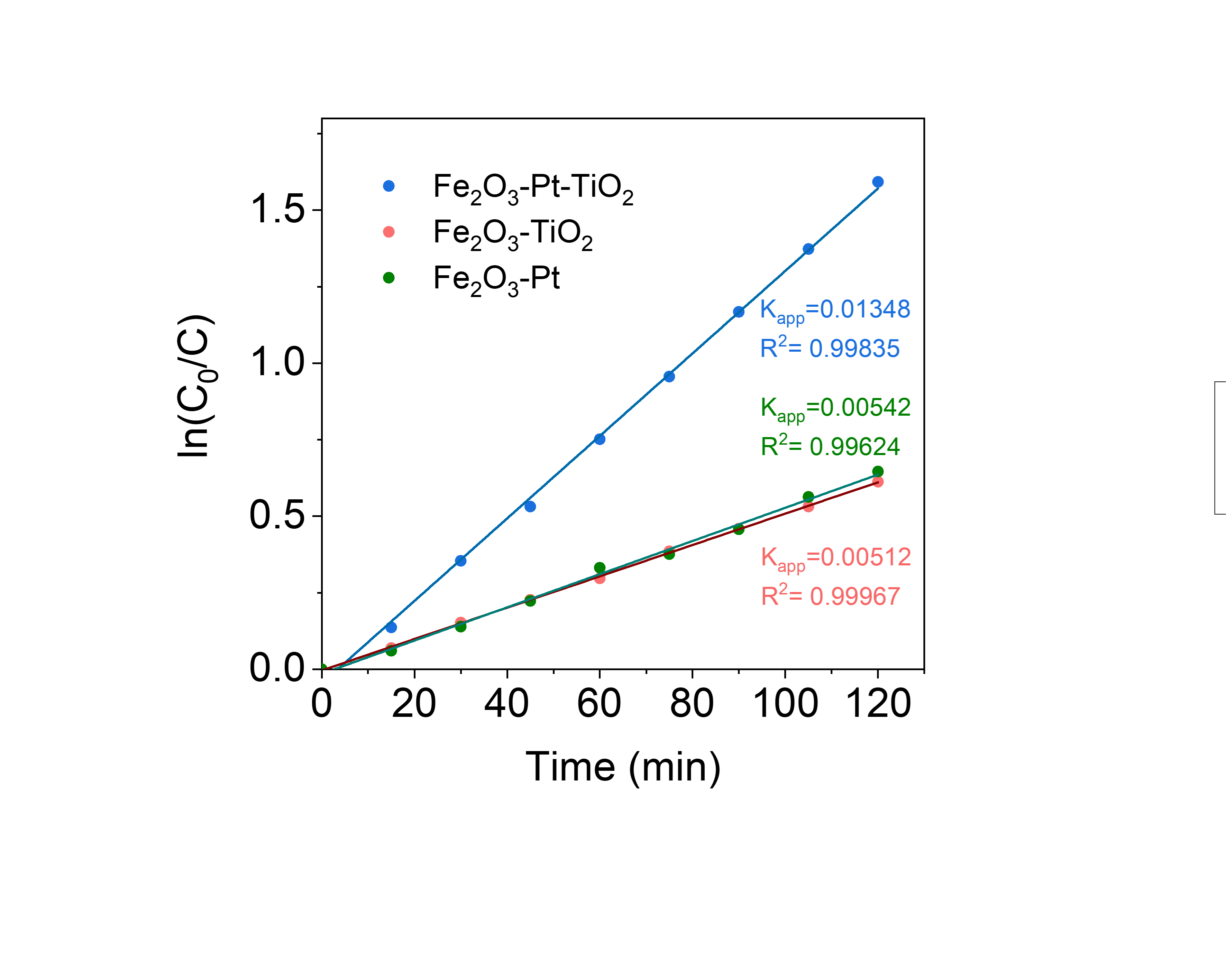


**Figure S12.** Pseudo-first-order kinetic fitting results of different samples.


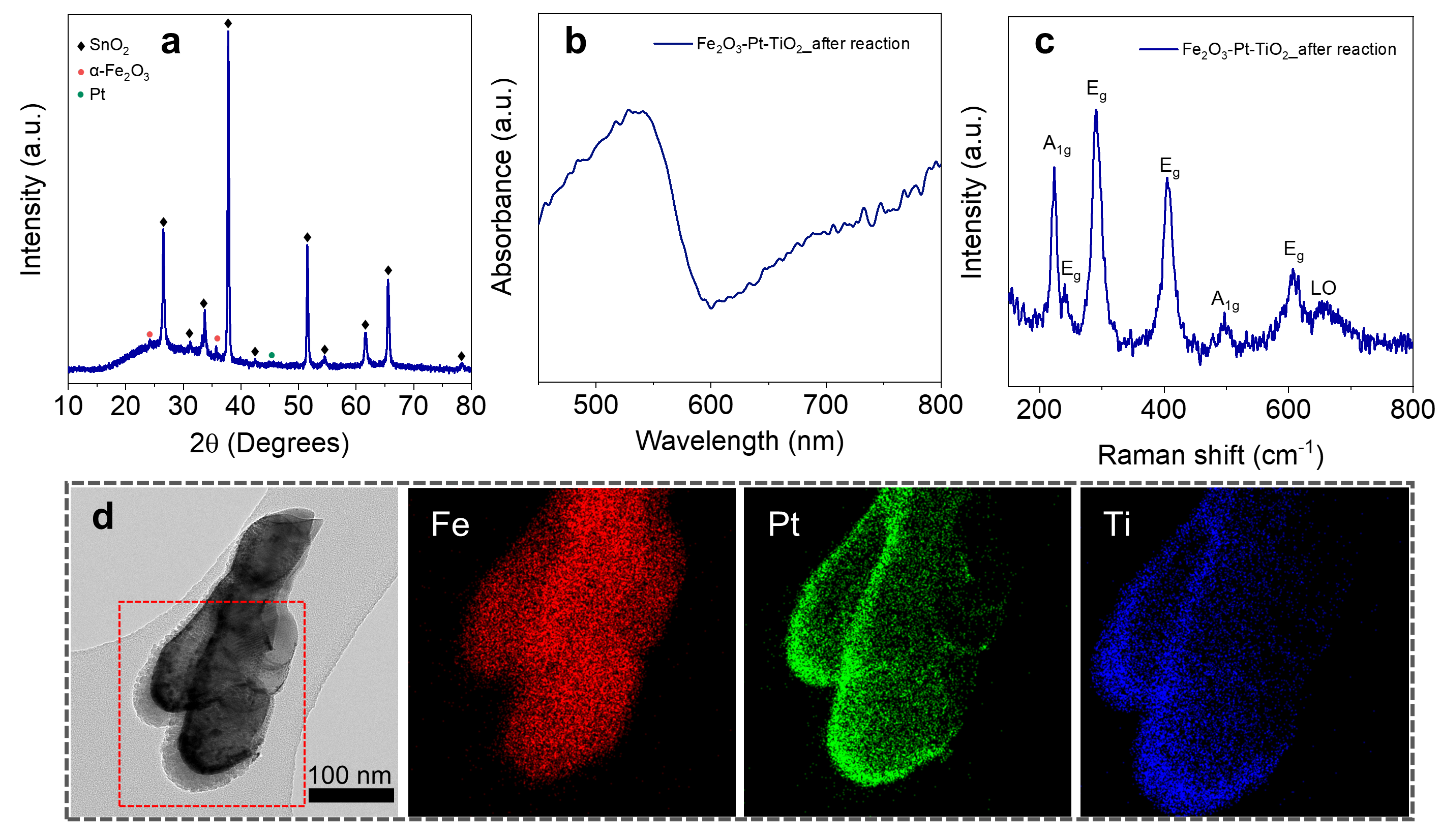


**Figure S13.** Characterization of Fe_2_O_3_-Pt-TiO_2_ after photocatalytic degradation of MB. (a) XRD pattern, (b) UV–vis absorption spectrum, (c) Raman spectrum, and (d) HRTEM image with corresponding EDX elemental mapping.


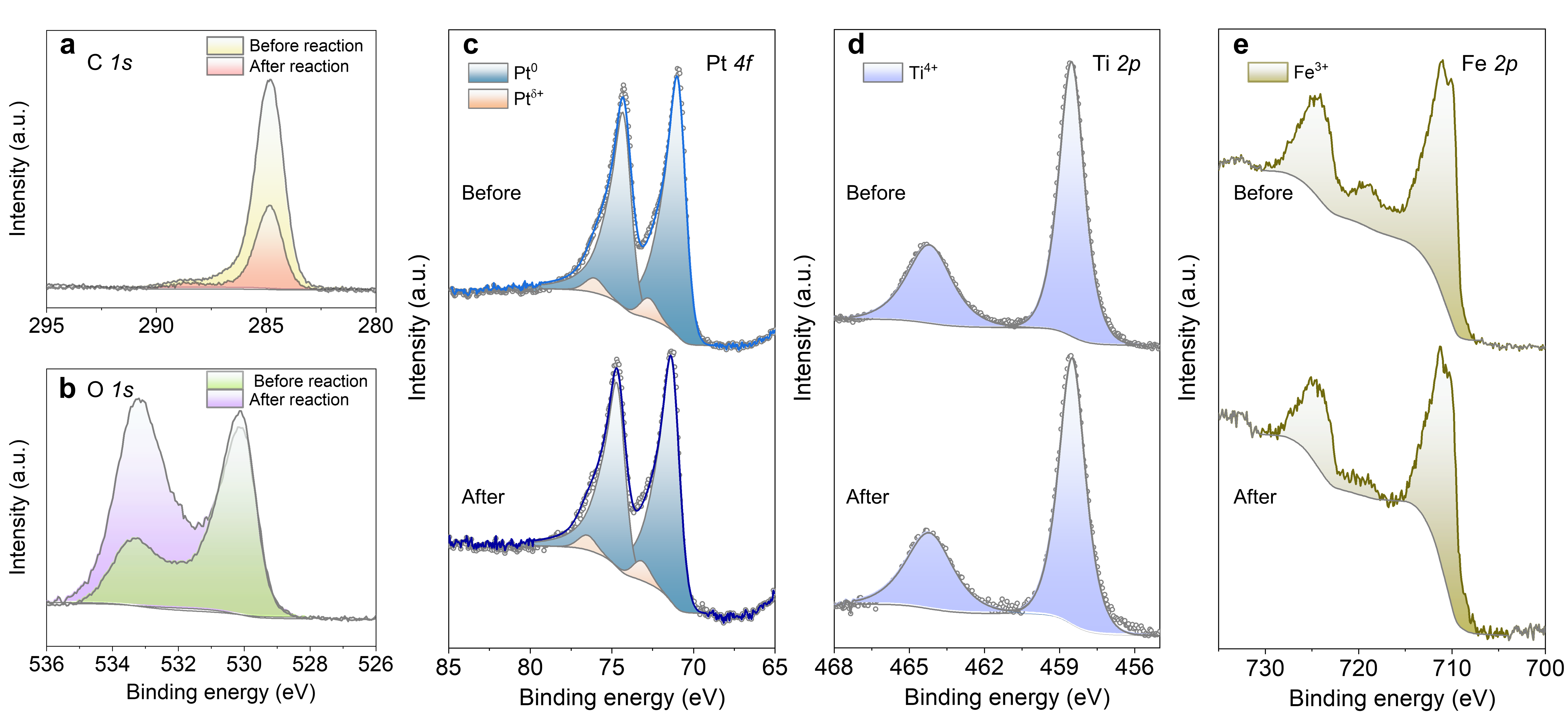


**Figure S14**. XPS spectra of the C *1s*, O *1s*, Pt *4f*, Ti *2p* and Fe *2p* lines obtained from Fe_2_O_3_-Pt-TiO_2_ nanomotors dispersed on doped silicon substrates before and after the photocatalytic MB degradation reaction. The binding energies of the samples after treatment have been shifted by -0.3 eV, due to some residual charging arising from the substrate. From the C *1s* lines, we observe a clear increase in carbon contamination for the sample exposed to the photocatalytic process, which induces a general decrease of the lines other than C *1s* (by a factor of 2, approximately). In the case of O *1s*, the spectra are normalized to the 530.1 eV peaks. The line corresponding to both Ti and Fe oxides (centered at 530.1 eV) clearly decreases after reaction. The line at approximately 533 eV corresponds to the native silicon oxide from the substrate. The curves from Pt *4f*, Ti *2p* and Fe *2p* have been shifted vertically and multiplied by the correction factor in order to be able to compare directly with the samples before reaction. The comparison verifies that the chemical states of Pt [Pt^0^], Ti [Ti^+4^] and Fe [Fe^+3^] remain essentially unchanged after the photocatalytic process. We also include a fit of the Pt *4f* line, where, apart from the dominant metallic features, the presence of oxidized species can be observed.

**Table S1.** Summary of recently reported Fe_2_O_3_-based photocatalysts for methylene blue (MB) degradation under light irradiation.

| Fe_2_O_3_ based-photocatalyst | Light source (light wavelength, intensity) | Initial MB concentration (ppm) | MB degradation efficiency | Photocatalyst dosage (mg/L) | Irradiation time (min) | Refs. |
| --- | --- | --- | --- | --- | --- | --- |
| Cu_2_V_2_O_7_/α-Fe_2_O_3_ | 300 W Xe lamp | 5 | 88% | 5 | 60 | ^16^ |
| ZnO/Fe_2_O_3_ | Green LED (520 nm, 0.5 mW cm^−2^) | 10 | 99.3% | 250 | 85 | ^17^ |
| TiO_2_/Fe_2_O_3_@nanographite | 400 W Metal halide lamp | 10 | 61% | 1250 | 120 | ^18^ |
| α-Fe_2_O_3_/g-C_3_N_4_ | 8 W UV light (365 nm) | 15 | 100% | 500 | 60 | ^19^ |
| rGO/g-C_3_N_4_/Fe_2_O_3_ | 40 W LED lamp (λ > 400 nm) | 10 | 97% | 500 | 120 | ^20^ |
| Fe_2_O_3_/ZnO | 300 W Xe lamp (λ > 420 nm, 3 mW cm^-2^) | 20 | 78% | 2000 | 210 | ^21^ |
| *α*-Fe_2_O_3_/ZnFe_2_O_4_/Mn_2_O_3_ | Sunlight | 5 | 95% | 1000 | 45 | ^22^ |
| Fe_2_O_3_-Pt-TiO_2_ | 460 nm blue LED (67 mW cm^-2^) | 10 | 91% | 33 | 120 | **This work** |

**Table S2.** BET analysis of α-Fe_2_O_3_ nanorods.

| Specific Surface area | 17.72 m^2^/g |
| --- | --- |
| Total Pore volume | 0.11 cm^3^/g |
| Average pore diameter | 24.71 nm |

**3. References**

(1) Ling, Y.; Wang, G.; Wheeler, D. A.; Zhang, J. Z.; Li, Y. Sn-Doped Hematite Nanostructures for Photoelectrochemical Water Splitting. *Nano Lett.* **2011**, *11*, 2119–2125. https://doi.org/10.1021/nl200708y.

(2) Fraxedas, J.; Zhang, K.; Sepúlveda, B.; Esplandiu, M. J.; De Andrés, X. G.; Llorca, J.; Pérez-Dieste, V.; Escudero, C. Water-Mediated Photo-Induced Reduction of Platinum Films. *J. Synchrotron Radiat.* **2019**, *26*, 1288–1293. https://doi.org/10.1107/S1600577519004685.

(3) Walton, J., Wincott, P., Fairley, N. & Carrick, A. Peak Fitting with CasaXPS: A Casa Pocket Book. 2010, Knutsford: Accolyte Science.

(4) Li, C.; Sampaoli, A.; Bäuerle, D.; Vera, M. B.; Peygourdi, G.; Willig, L.; Kennis, J. T. M.; Liguori, N.; 1ICFO. Real-Time Tracking of Ground- and Excited-State Responses of Photoactive Molecules to a Change of Microenvironment, from Femtoseconds to Milliseconds. *ChemRxiv* **2024**.

(5) Müller, C.; Pascher, T.; Eriksson, A.; Chabera, P.; Uhlig, J. KiMoPack: A Python Package for Kinetic Modeling of the Chemical Mechanism. *J. Phys. Chem. A* **2022**, *126*, 4087–4099. https://doi.org/10.1021/acs.jpca.2c00907.

(6) Lohaus, C.; Klein, A.; Jaegermann, W. Limitation of Fermi Level Shifts by Polaron Defect States in Hematite Photoelectrodes. *Nat. Commun.* **2018**, *9*, 4309. https://doi.org/10.1038/s41467-018-06838-2.

(7) Fan, H.; Yang, Z.; Ren, X.; Yin, M.; Gao, F.; Liu, S. F. Band Alignment of TiO_2_/FTO Interface Determined by X-Ray Photoelectron Spectroscopy: Effect of Annealing. *AIP Adv.* **2016**, *6*, 015314. https://doi.org/10.1063/1.4941040.

(8) Kraushofer, F.; Jakub, Z.; Bichler, M.; Hulva, J.; Drmota, P.; Weinold, M.; Schmid, M.; Setvin, M.; Diebold, U.; Blaha, P.; Parkinson, G. S. Atomic-Scale Structure of the Hematite α-Fe_2_O_3_(11-02) “r-Cut” Surface. *J. Phys. Chem. C* **2018**, *122*, 1657–1669. https://doi.org/10.1021/acs.jpcc.7b10515.

(9) Kashiwaya, S.; Morasch, J.; Streibel, V.; Toupance, T.; Jaegermann, W.; Klein, A. The Work Function of TiO_2_. *Surfaces* **2018**, *1*, 73–89. https://doi.org/10.3390/surfaces1010007.

(10) Huang, Z.; Lin, Y.; Xiang, X.; Rodríguez-Córdoba, W.; McDonald, K. J.; Hagen, K. S.; Choi, K. S.; Brunschwig, B. S.; Musaev, D. G.; Hill, C. L.; Wang, D.; Lian, T. In Situ Probe of Photocarrier Dynamics in Water-Splitting Hematite (α-Fe_2_O_3_) Electrodes. *Energy Environ. Sci.* **2012**, *5*, 8923–8926. https://doi.org/10.1039/c2ee22681b.

(11) Barroso, M.; Mesa, C. A.; Pendlebury, S. R.; Cowan, A. J.; Hisatomi, T.; Sivula, K.; Graẗzel, M.; Klug, D. R.; Durrant, J. R. Dynamics of Photogenerated Holes in Surface Modified α-Fe_2_O_3_ Photoanodes for Solar Water Splitting. *Proc. Natl. Acad. Sci. U. S. A.* **2012**, *109*, 15640–15645. https://doi.org/10.1073/pnas.1118326109.

(12) Barroso, M.; Pendlebury, S. R.; Cowan, A. J.; Durrant, J. R. Charge Carrier Trapping, Recombination and Transfer in Hematite (α-Fe_2_O_3_) Water Splitting Photoanodes. *Chem. Sci.* **2013**, *4*, 2724–2734. https://doi.org/10.1039/c3sc50496d.

(13) Joly, A. G.; Williams, J. R.; Chambers, S. A.; Xiong, G.; Hess, W. P.; Laman, D. M. Carrier Dynamics in α-Fe_2_O_3_ (0001) Thin Films and Single Crystals Probed by Femtosecond Transient Absorption and Reflectivity. *J. Appl. Phys.* **2006**, *99*. https://doi.org/10.1063/1.2177426.

(14) Hayes, D.; Hadt, R. G.; Emery, J. D.; Cordones, A. A.; Martinson, A. B. F.; Shelby, M. L.; Fransted, K. A.; Dahlberg, P. D.; Hong, J.; Zhang, X.; Kong, Q.; Schoenlein, R. W.; Chen, L. X. Electronic and Nuclear Contributions to Time-Resolved Optical and X-Ray Absorption Spectra of Hematite and Insights into Photoelectrochemical Performance. *Energy Environ. Sci.* **2016**, *9*, 3754–3769. https://doi.org/10.1039/c6ee02266a.

(15) Pendlebury, S. R.; Barroso, M.; Cowan, A. J.; Sivula, K.; Tang, J.; Grätzel, M.; Klug, D.; Durrant, J. R. Dynamics of Photogenerated Holes in Nanocrystalline α-Fe_2_O_3_ Electrodes for Water Oxidation Probed by Transient Absorption Spectroscopy. *Chem. Commun.* **2011**, *47*, 716–718. https://doi.org/10.1039/c0cc03627g.

(16) Li, F.; Li, X.; Tong, S.; Wu, J.; Zhou, T.; Liu, Y.; Zhang, J. S-Scheme Heterojunction Cu_2_V_2_O_7_/α-Fe_2_O_3_ Nanosheets with Effective Photocatalytic Activity toward Organic Pollutant Degradation. *Nano Energy* **2023**, *117*, 108849. https://doi.org/10.1016/j.nanoen.2023.108849.

(17) Dien, N. D.; Pham, T. T. H.; Vu, X. H.; Xuan, V. T.; Nguyen, T. T. T.; Trang, T. T.; Van Hao, N.; Nga, P. T.; Kim Chi, T. T.; Giang, T. T. H.; Toan, N. D. High Photocatalytic Efficiency of a ZnO Nanoplate/Fe_2_O_3_ Nanospindle Hybrid Using Visible Light for Methylene Blue Degradation. *RSC Adv.* **2024**, *14*, 28244–28259. https://doi.org/10.1039/d4ra04230a.

(18) Mensah, K.; Shokry, H.; Elkady, M.; Hawash, H. B.; Samy, M. Enhanced Photocatalytic Degradation of Dyes Using a Novel Waste Toner-Based TiO_2_/Fe_2_O_3_@nanographite Nanohybrid: A Sustainable Approach. *Water Sci. Eng.* **2024**, *17*, 226–235. https://doi.org/10.1016/j.wse.2024.01.005.

(19) Galán-González, A.; Fernández, I.; Zaluzec, N. J.; Cambré, S.; Arenal, R.; Benito, A. M.; Maser, W. K. Fabrication of α-Fe_2_O_3_ Nanoparticles/g-C_3_N_4_ Direct Z-Scheme Heterojunction of Durable Photocatalytic Activity. *ACS Appl. Nano Mater.* **2025**, *8*, 9364–9375. https://doi.org/10.1021/acsanm.5c00991.

(20) Palanivel, B.; Hossain, M. S.; Raghu, M. S.; Kumar, K. Y.; Macadangdang, R. R.; Ubaidullah, M.; Prakash, C.; Bommireddy, P. R.; Park, S. H. Green Synthesis of Fe_2_O_3_ Deposited G-C_3_N_4_: Addition of RGO Promoted Z-Scheme Ternary Heterojunction for Efficient Photocatalytic Degradation and H_2_ Evolution Reaction. *Mater. Res. Bull.* **2023**, *162*, 112177. https://doi.org/10.1016/j.materresbull.2023.112177.

(21) Lin, Q.; Zhang, R.; Zhang, X.; Li, S.; Dai, J.; Li, S.; Wang, Z.; Liang, D.; Fu, H.; Zhang, X. Electrospun Fiber Membranes of Fe_2_O_3_/ZnO with High Photocatalytic Activity for Wastewater Treatment Application under Visible Light Irradiation. *Catal. Letters* **2025**, *155*, 67. https://doi.org/10.1007/s10562-024-04905-6.

(22) Yang, W.; Xu, C.; Lyu, Y.; Lan, Z.; Li, J.; Ng, D. H. L. Hierarchical Hollow α-Fe_2_O_3_/ZnFe_2_O_4_/Mn_2_O_3_ Janus Micromotors as Dynamic and Efficient Microcleaners for Enhanced Photo-Fenton Elimination of Organic Pollutants. *Chemosphere* **2023**, *338*, 139530. https://doi.org/10.1016/j.chemosphere.2023.139530.
